# Functional data analysis for longitudinal data with informative observation times

**DOI:** 10.1111/biom.13646

**Published:** 2022-03-28

**Authors:** Caleb Weaver, Luo Xiao, Wenbin Lu

**Affiliations:** Department of Statistics, North Carolina State University, Raleigh, North Carolina, USA

**Keywords:** functional data analysis, informative observation times, longitudinal data, penalized splines

## Abstract

In functional data analysis for longitudinal data, the observation process is typically assumed to be noninformative, which is often violated in real applications. Thus, methods that fail to account for the dependence between observation times and longitudinal outcomes may result in biased estimation. For longitudinal data with informative observation times, we find that under a general class of shared random effect models, a commonly used functional data method may lead to inconsistent model estimation while another functional data method results in consistent and even rate-optimal estimation. Indeed, we show that the mean function can be estimated appropriately via penalized splines and that the covariance function can be estimated appropriately via penalized tensor-product splines, both with specific choices of parameters. For the proposed method, theoretical results are provided, and simulation studies and a real data analysis are conducted to demonstrate its performance.

## 1 ∣ INTRODUCTION

In functional data analysis for longitudinal data, the observation process is typically assumed to be noninformative, that is, itis assumed tobe independent of longitudinal outcomes. However, this assumption is often violated in real applications. For example, in a longitudinal study, the frequency and timing of a subject’s observations may be correlated with the values of longitudinal outcomes. Data collected in such a setting are referred to as longitudinal data with informative observation times.

**FIGURE 1:**
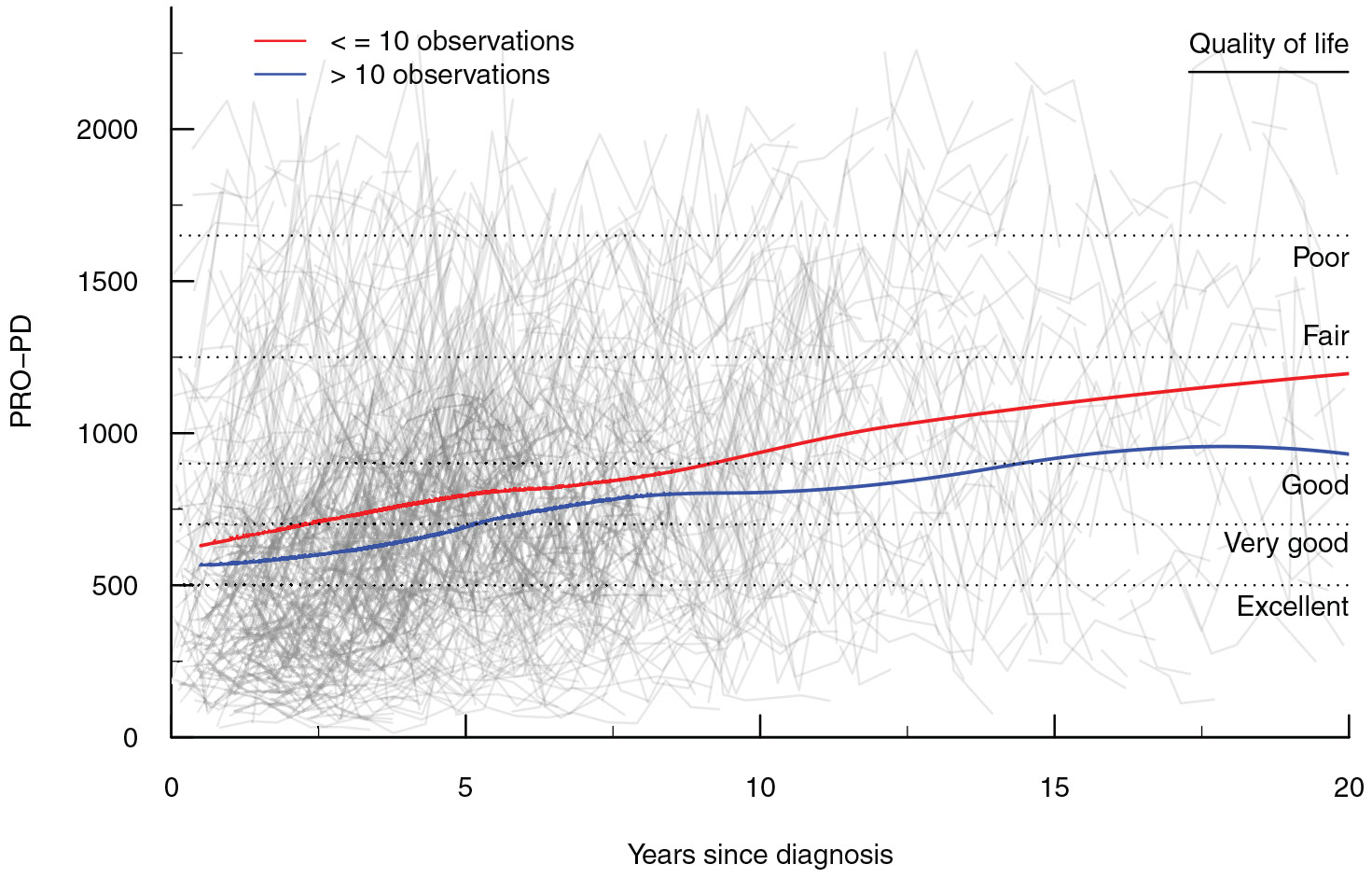
The 371 trajectories of symptom severity along with smoothed mean profiles of subjects with less than or equal to 10 observations and of subjects with more than 10 observations. Reference lines indicate the average PRO-PD of subjects answering the prompt “In general, would you say your quality of life is:” with either “poor,” “fair,” “good,” “very good,” or “excellent.”

A motivating example is a longitudinal data set of trajectories of patient-reported symptom severity of Parkinson’s disease (Mischley et al., 2017). The data consist of self-reported severity of each of 33 symptoms on a scale of 0–100 for 371 patients, with the higher number reflecting greater symptom severity. The sum of these symptoms generates the total patient-reported outcome in Parkinson’s disease (PRO-PD) score. The number of observations for each patient ranges from 2 to 38, and observations are made on a voluntary basis at varying times since a patient’s initial diagnosis. Patients entered the study at arbitrary times since diagnosis. There are no instances of dropout, and we only consider data observed less than 20 years following any patient’s initial diagnosis. Figure 1 illustrates the 371 trajectories along with smoothed group-mean profiles of subjects with 10 or fewer observations and of subjects with more than 10 observations in relation to patient-reported quality of life (Cella et al., 2010). The average (standard error) PRO-PD score of subjects with 10 or fewer observations is 828.3 (16.5), and the average PRO-PD score of subjects with more than 10 observations is 752.4 (13.2). It is apparent that subjects reporting lower symptom severity tend to report more frequent observations, which provides evidence against the assumption of noninformative observation times.

It is well understood that standard methods for longitudinal data that ignore the informative observation processes may result in bias in parameter estimation. Hence, a number of methods have been proposed for the situation in which longitudinal outcomes are correlated with observation times (Lin et al., 2004; Sun et al., 2005, 2007, 2012; Liang et al., 2009; Zhu et al., 2011; Cai et al., 2012; Song et al., 2012; Zhao et al., 2012; Fang et al., 2016). Among them, a commonly used modeling strategy is to consider shared random effects models (eg, Sun et al., 2005; Liang et al., 2009). Such models can allow flexible dependence between longitudinal outcomes and observation times.

However, existing literature for longitudinal data with informative observation times mostly focuses on the estimation of an unspecified smooth mean function or integrated mean function. In the analysis of longitudinal data, besides the mean function, it might also be interesting to consider a nonparametric covariance function, which captures the patterns of subject-specific trajectories. A popular nonparametric modeling approach for estimating both mean and covariance functions of longitudinal data is via methods of functional data analysis (Ramsay & Silverman, 2006), in which one views the values of a longitudinal trajectory as observations from a random smooth function; see Guo (2002) and Yao et al. (2003, 2005). The advantage of functional data methods compared to traditional parametric longitudinal methods is that they allow for unspecified mean/covariance functions, which may better capture complex variation patterns of subject-specific trajectories.

Theoretical properties of functional data analysis have been well studied in several papers (Li & Hsing, 2010; Cai & Yuan, 2011; Zhang & Wang, 2016; Xiao, 2020). In particular, Zhang and Wang (2016) and Xiao (2020) considered general weighting schemes for aggregating multiple observations of each subject in the estimation of mean and covariance functions of functional data. The theories therein assume that the observation times are either fixed or randomly sampled and independent from the longitudinal outcomes. Hence, existing theories are not directly applicable to settings in which the observation process is informative. Nevertheless, it might be helpful to clarify that, as shall be seen by our model specification, we consider longitudinal data where each subject only has only a finite number of observations, and hence the sparse to dense phase transition of functional data in terms of the number of observations per subject as those in Cai and Yuan (2011) and other works is not relevant here.

In this paper, we consider functional data methods for longitudinal data with informative observation times. We use a heuristic argument to show that a commonly used functional data method may be inconsistent for such data. Using the same argument, we identify one particular functional data method that could give consistent estimation under the considered shared random effect model. The difference in these methods lies in how the multiple observations of each subject are aggregated to formulate the weighted least square estimation. This contrasts dramatically with existing functional data theories, which found that both methods lead to consistent estimation. For the suggested method with penalized splines, we derive the corresponding rates of convergence, which are either optimal or the best in the literature. It is worthy of pointing out that the theoretic derivation is far from a straightforward application of existing theoretic results which consider noninformative observation times while the theory in the paper has to handle informative time points. Simulation studies demonstrate the desired performance of the suggested method and the inconsistent behavior of the aforementioned commonly used method in functional data analysis. Finally, the suggested method is applied to the motivating data and we conclude the paper with some discussion.

## 2 ∣ METHODS

### 2.1 ∣ Model specification

Let $Y_{i}(t)$ be subject i’s underlying longitudinal outcome (i=1,…,n) at time t∈𝒯 where 𝒯 is a compact time interval. Without loss of generality, let 𝒯=[0,1]. The observations $Y_{ij}=Y_{i}(T_{ij})$ of subject i are taken at time points $T_{i1}<T_{i2}<\ldots <T_{im_{i}}$. Let $C_{i}\in 𝒯$ denote the censoring time of the ith subject. The cumulative number of observations for the ith subject by time t is $N_{i}(t)=N_{i}^{*}(t\;\wedge \;C_{i})$, where $N_{i}^{*}(t)=\sum_{j\geq 1}I(T_{ij}\leq t)$. Here, I is the indicator function and s∧t denotes the minimum of s and t. The observed values of $Y_{i}(t)$ are obtained at the jump points of $N_{i}(t)$. Consider the functional data model

(1) $$Y_{i}(t)=\mu^{*}(t,Z_{i})+X_{i}(t)+\epsilon_{i}(t),$$

where $\mu^{*}(\cdot ,Z_{i})$ is a conditional mean function parameterized by the latent frailty variable $Z_{i}$, $X_{i}(\cdot )$ is a zero-mean random function that models subject i’s smooth deviation from the mean, and $\epsilon_{i}(\cdot )$ is zero-mean white noise with finite variance $\sigma_{\epsilon }^{2}$. Define the marginal mean function $\mu (t)=E\{\mu^{*}(t,Z_{i})\}$, the covariance function of the conditional mean functions $\sigma_{\mu^{*}}(s,t)=\mathrm{cov}\{\mu^{*}(s,Z_{i}),\mu^{*}(t,Z_{i})\}$, and the covariance function of the random subject-specific functions $\sigma_{X}(s,t)=\mathrm{cov}\{X_{i}(s),X_{i}(t)\}$. Then, the responses can be considered as observations from a random function with mean function μ(t) and covariance function $\sigma (s,t)=\sigma_{\mu^{*}}(s,t)+\sigma_{X}(s,t)$, contaminated with measurement error.

As commonly considered in shared random effects models (eg, Liang et al., 2009), we make the following assumptions: (1) censoring time $C_{i}$ is independent of the observation times $\left\{T_{ij},j\geq 1\right\}$ and longitudinal outcomes $\left\{Y_{i}(T_{ij}),j\geq 1\right\}$; (2) given the frailty variable $Z_{i}$, the observation process $N_{i}^{*}(\cdot )$ is independent of the longitudinal outcomes $\left\{Y_{i}(T_{ij}),j\geq 1\right\}$; and (3) given the frailty variable $Z_{i}$, the observation process $N_{i}^{*}(t)$ follows a Poisson process with the intensity function $\lambda (t,Z_{i})=Z_{i}\lambda_{0}(t)$, where $\lambda_{0}(t)$ is an unspecified intensity function. Define $\Lambda_{0}(t)=\int_{0}^{t}\lambda_{0}(s)\;ds$, the baseline cumulative intensity function.

The dependence between longitudinal outcomes and observation times is induced by the latent frailty variable $Z_{i}$, but the form and magnitude of the dependence is left completely unspecified for flexibility. To simplify theoretical analysis, we allow $m_{i}$ to be 0. In formulas and equations involving $m_{i}^{-1}$, the term $m_{i}^{-1}$ should always be interpreted as $m_{i}^{-1}I(m_{i}\neq 0)$, which equals 0 if $m_{i}=0$.

### 2.2 ∣ Estimation of mean function

The mean function μ(t) is approximated by a spline function $B^{T}(t)\theta$ where $B(t)={\left\{B_{1}(t),\ldots ,B_{K}(t)\right\}}^{T}\in R^{K}$ is a vector of rth order B-spline basis functions constructed from equally spaced knots in 𝒯, and θ is a vector of coefficients. The coefficient vector θ is estimated via the minimization

(2) $$\hat{\theta }=\text{arg}\;\underset{\theta }{\text{min}}\left[\sum_{i=1}^{n}\sum_{j=1}^{m_{i}}w_{i}{\left\{Y_{ij}-B^{T}(T_{ij})\theta \right\}}^{2}+\lambda \theta^{T}P\theta \right],$$

where $w_{i}s$ are nonnegative weights that may depend on $m_{i}$ and $C_{i}$, λ is a smoothing parameter that balances fit and smoothness, and P is a penalty matrix such that $\theta^{T}P\theta$ equals the squared sum of the qth order consecutive differences of θ as in Eilers and Marx (1996). The mean function is then estimated by $\hat{\mu }(t)=B^{T}(t)\hat{\theta }$.

A common choice of the weights is $w_{i}=1$, which means that each observation has equal weight in forming the least squares in (2). We will denote this choice of weights by “OBS,” following Zhang and Wang (2016) and Xiao (2020). To our knowledge, this is the most often used choice for formulating the least squares in functional data analysis. Another choice of weights is to $w_{i}=m_{i}^{-1}$, which implies that the set of observations from each subject has equal weight (eg, Li & Hsing, 2010). We will denote this choice of weights by “SUBJ.” Both choices of weights could lead to consistent and even rate-optimal estimation in functional data analysis as shown in Zhang and Wang (2016) and Xiao (2020) for functional data with noninformative observation times. However, in what follows, we shall heuristically derive different weighting schemes will affect estimation for functional data with informative observation times.

Let ${𝒜}_{i}(t)=w_{i}\{Y_{i}(t)-\mu_{n}(t)\}\;dN_{i}(t)$, where $\mu_{n}(t)$ is the solution to the estimating equation $\sum_{i=1}^{n}{𝒜}_{i}(t)=0$. Then, we have

(3) $$\mu_{n}(t)=\frac{\sum_{i=1}^{n}w_{i}Y_{i}(t)dN_{i}(t)}{\sum_{i=1}^{n}w_{i}dN_{i}(t)}.$$

As shown in Liang et al. (2009),

$$E\{dN_{i}(t)\;|\;m_{i},C_{i}\}=I(C_{i}\geq t)m_{i}\frac{d\Lambda_{0}(t)}{\Lambda_{0}(C_{i})}.$$

Thus,

$$E\{w_{i}Y_{i}(t)dN_{i}(t)\;|\;C_{i}\}\;\;=E[w_{i}E\{Y_{i}(t)\;dN_{i}(t)\;|\;m_{i},C_{i}\}\;|\;C_{i}]\;\;=E\left[w_{i}I(C_{i}\geq t)m_{i}\frac{d\Lambda_{0}(t)}{\Lambda_{0}(C_{i})}E\{\mu^{*}(t,Z_{i})\;|\;m_{i},C_{i}\}\;|\;C_{i}\right]\;\;=I(C_{i}\geq t)\frac{d\Lambda_{0}(t)}{\Lambda_{0}(C_{i}}E\{\mu^{*}(t,Z_{i})w_{i}m_{i}\;|\;C_{i}\}.$$

Similarly, we derive that

$$E\{w_{i}\;dN_{i}(t)\;|\;C_{i}\}=I(C_{i}\geq t)\frac{d\Lambda_{0}(t)}{\Lambda_{0}(C_{i})}E(w_{i}m_{i}\;|\;C_{i}).$$

It follows by Equation (3) that as n increases to infinity, $\mu_{n}(t)$ converges to

(4) $$\mu^{o}(t)\equiv \frac{E\left[\frac{I(C_{i}\geq t)}{\Lambda_{0}(C_{i})}E\{\mu^{*}(t,Z_{i})w_{i}m_{i}\;|\;C_{i}\}\right]}{E\left\{\frac{I(C_{i}\geq t)}{\Lambda_{0}(C_{i})}E(w_{i}m_{i}\;|\;C_{i})\right\}}.$$

We now evaluate expression (4) with either the OBS weights or the SUBJ weights. First, consider the SUBJ weights for which $w_{i}=m_{i}^{-1}$. Then $w_{i}m_{i}=1$. Because $Z_{i}$ is independent of $C_{i}$, $\mu^{o}(t)=E\{\mu^{*}(t,Z_{i})\;|\;C_{i}\}=E\{\mu^{*}(t,Z_{i})\}=\mu (t)$. Hence, for the SUBJ weights, $\mu_{n}(t)$ converges to μ(t) as desired. Second, consider the OBS weights for which $w_{i}=1$. In general, in this case $\mu^{o}(t)$ in (4) is different from μ(t). To give an example, consider the simulation setting in Section 4, where we let $\mu^{*}(t,Z)=Z(t^{2}+2∕3)$, $Z=(1-\rho )+\rho Z^{*}$ for some 0<ρ<1, and $Z^{*}$ is from a distribution with mean 1 and variance $\sigma_{Z^{*}}^{2}$. In addition, m follows a Poisson distribution with mean $10Z\Lambda_{0}(C)$. Here, (Z,m,C) has the same distribution as ($Z_{i},m_{i},C_{i}$). Then we derive that $E(m\;|\;C)=10E\{Z\Lambda_{0}(C)\;|\;C\}=10\Lambda_{0}(C)$, $\mu^{*}(t,Z)m=Zm(t^{2}+2∕3)$, and $E\{\mu^{*}(t,Z)m\;|\;c\}=(t^{2}+2∕3)E(Zm\;|\;C)=10(t^{2}+2∕3)E\{Z^{2}\Lambda_{0}(C)\;|\;C\}=10\Lambda_{0}(C)(t^{2}+2∕3)(1+\rho^{2}\sigma_{Z^{*}}^{2})$. It follows that $\mu^{o}(t)=(t^{2}+2∕3)(1+\rho^{2}\sigma_{Z^{*}}^{2})$, which differs from $\mu (t)=t^{2}+2∕3$. Indeed, our simulation study in Section 4 will provide empirical confirmation of the above derivations. Another general example is when $C_{i}=1$ for all i, that is, no censoring. Then it is easy to see that (4) reduces to $\mu^{o}(t)=E\left\{\mu^{*}(t,Z)m\right\}∕E(m)$, which differs from $\mu (t)=E\{\mu^{*}(t,Z)\}$ unless $\mu^{*}(t,Z)$ and m are uncorrelated.

The above derivations show that the OBS weights lead to inconsistent estimation of the mean function while in contrary the SUBJ weights might give consistent estimation. This differs from traditional functional data analysis, where both OBS and SUBJ weights will lead to consistent estimation (Zhang & Wang, 2016; Xiao, 2020). Thus, for functional data with informative observation times under the shared random effects model, we propose to use the SUBJ weights. Accordingly, we shall rigorously derive the rate of convergence of the mean function estimation in Section 3.

### 2.3 ∣ Estimation of covariance function

Let $\hat{\mu }(t)$ be the estimate of the mean function using the SUBJ weights. Let ${\tilde{e}}_{ij}=Y_{ij}-\hat{\mu }(T_{ij})$ and define the auxiliary variables ${\tilde{\sigma }}_{ij_{1}j_{2}}={\tilde{e}}_{ij_{1}}{\tilde{e}}_{ij_{2}}$, which is an empirical estimate of $\sigma (T_{ij_{1}},T_{ij_{2}})$. Then $\left\{(T_{ij_{1}},T_{ij_{2}},{\tilde{\sigma }}_{ij_{1}j_{2}}):1\leq j_{1}\neq j_{2}\leq m_{i},i=1,\ldots ,n\right\}$ forms a data set for estimating the covariance function.

The covariance function σ(s,t) is approximated by a tensor-product spline, $H(s,t)=\sum_{1\leq k,l\leq K}\theta_{kl}B_{k}(s)B_{l}(t)$, where $\Theta ={\left(\theta_{kl}\right)}_{1\leq k\leq K,1\leq l\leq K}$ is a coefficient matrix. To simplify notation, we use the same univariate B-splines as in the mean function estimation. Since the covariance function is symmetric, it is natural to impose the constraint that $\Theta =\Theta^{T}$, which ensures that H(s,t)=H(t,s). Let vec(⋅) be the vectorization operator, which is invertible, and define $\theta_{\sigma }=\mathrm{vec}(\Theta )$. Let B(s,t)=B(t)⊗B(s), where ⊗ is the Kronecker product. Then, $H(s,t)=B^{T}(s,t)\theta_{\sigma }$. The coefficient matrix Θ is estimated via the minimization

(5) $$\hat{\Theta }=\text{arg}\;\underset{\Theta \;:\Theta =\Theta^{T}}{\text{min}}\;\sum_{i=1}^{n}\left[v_{i}\sum_{1\leq j_{1}\neq j_{2}\leq m_{i}}{\left\{{\tilde{\sigma }}_{ij_{1}j_{2}}-H(T_{ij_{1}},T_{ij_{2}})\right\}}^{2}\right]\;\;+\lambda K^{-1}\theta_{\sigma }^{T}(I_{K}⊗P)\theta_{\sigma },$$

where $v_{i}s$ are nonnegative weights, $\lambda K^{-1}$ is a smoothing parameter, and P is the same penalty matrix for mean function estimation. We use the same notation λ as in the mean function estimation to simplify the notation in theoretical analysis. The above method was proposed in Xiao et al. (2018) and can be shown to be a special case of the bivariate tensor-product P-splines in Eilers and Marx (2003).

Let ${\hat{\theta }}_{\sigma }=\mathrm{vec}(\hat{\Theta })$. Then, the covariance function σ(s,t) is estimated by $\hat{\sigma }(s,t)=B^{T}(s,t){\hat{\theta }}_{\sigma }$. As in the mean function estimation, we consider two choices of weights. We will denote the choice of $v_{i}=1$ by “OBS” and the choice of $v_{i}={\left\{m_{i}(m_{i}-1)\right\}}^{-1}$ by “SUBJ.” Similar to the mean function estimation, the former weight implies each auxiliary variable carries the same weight in forming the weighted least squares while the latter implies the set of auxiliary variables from each subject carries the same weight. Then, by similar arguments (omitted) as in the mean function estimation, it can be shown that the SUBJ weights should be adopted. And we shall also derive rates of convergence of the above proposed estimator with the SUBJ weights.

## 3 ∣ THEORETICAL PROPERTIES

### 3.1 ∣ Notation

For a univariate function g over 𝒯, ‖g‖ denotes its supreme norm. The same notation is also used for a bivariate function. We also use $g^{\left(1\right)}$ to denote the derivative of a function g. For two scalars, let a∧b=min(a,b) and a∨b=max(a,b). We let $\underline{m}=\{m_{i},1\leq i\leq n\}$ and $\underline{C}=\{C_{i},1\leq i\leq n\}$.

### 3.2 ∣ Asymptotic properties of mean function estimator

**Assumption 1.** The nonnegative latent variables $Z_{i}(1\leq i\leq n)$ are independent and identically distributed and satisfy, for any positive constant c>0, $0<E(Z_{1}e^{-cZ_{1}})<\infty$.

**Assumption 2.** The censoring times $C_{i}(1\leq i\leq n)$ are independent and identically distributed.

**Assumption 3.** The baseline hazard function $\Lambda_{0}(t)$ has a positive and continuous density function on 𝒯.

**Assumption 4.** Let $\rho_{1}(t)=E\{\Lambda_{0}^{\left(1\right)}(t\wedge C_{1})∕\Lambda_{0}(C_{1})\}$ and $\rho_{2}(s,t)=E\Lambda_{0}^{\left(1\right)}(t\wedge C_{1})\Lambda_{0}^{\left(1\right)}(s\wedge C_{1})∕\Lambda_{0}^{2}(C_{1})\}$. Then, $\rho_{1}$ and $\rho_{2}$ are continuous and bounded away from 0 and infinity, over 𝒯 and ${𝒯}^{2}$, respectively.

**Assumption 5.** (a) $\|\sigma \|<\infty ;(b)\sigma_{\epsilon }^{2}<\infty$.

**Assumption 6.** (a) The number of basis functions K satisfies $K\geq n^{\delta_{1}}$ for some constant $\delta_{1}>0$ and K=o(n); (b) The smoothing parameter λ satisfies $\lambda =o(n^{-\delta_{2}})$ for some constant $\delta_{2}>0$; (c) $\log\;n∕n=o(K^{-4})$.

We now discuss Assumption 4. First, $\rho_{1}(t)$ is the density function of the theoretical counterpart of the empirical distribution of the observed time points. In nonparametric regression, it is usually required this function is bounded away from 0 and infinity. When there are no censoring, that is, $C_{i}=1$, then $\rho_{1}(t)=\Lambda_{0}^{\left(1\right)}(t)$ and $\rho_{2}(s,t)=\Lambda_{0}^{\left(1\right)}(s)\Lambda_{0}^{\left(1\right)}(t)$. Because of Assumption 3, Assumption 4 will always hold. When there are censoring, we could show that one generating distribution for $C_{i}$ in the simulation study also ensures that Assumption 4 holds.

For theoretical results, we shall focus on the SUBJ weights and scale the weights $w_{i}$ with $w_{i}={\left(nm_{i}\right)}^{-1}$ so that $\sum_{i=1}^{n}w_{i}m_{i}=1$. The notation r denotes the order of splines and q denotes the order of penalty. Let $h=K^{-1}$ and $h_{e}=h\vee \lambda^{1∕(2q)}$. By Xiao (2019), $h_{e}$ plays the role of bandwidth parameter for penalized splines.

**Theorem 1.** Suppose that Assumptions 1-6 hold. If $\mu \in {𝒞}^{p}(𝒯)$ with q≤p∧r, then

$$E\left[\int_{𝒯}{\left\{\hat{\mu }(t)-\mu (t)\right\}}^{2}\;dt|\underline{m},\underline{C}\right]\;\;=O(h^{2r})+o(h^{2p})+O\left(\lambda^{2}h_{e}^{-2q}\right)+O(n^{-1}\;h_{e}^{-1}),\;a.s.$$

of Theorem 1. The proof of Theorem 3.3 in Xiao (2020) can be adapted if Lemmas A.7, A.8, and A.9 in Xiao (2020) hold almost surely, which are given in Lemma 1 and proved in Section A of the Supporting Information. These lemmas deal with empirical distributions of the observed time points, which are informative, and hence different proofs are required.

The derived $L_{2}$ convergence rate in Theorem 1 is the same as the those in Xiao (2020) for the scenario with a finite number observations per subject and randomly observed time points. Moreover, as shown in Claeskens et al. (2009), Xiao (2019), and Huang and Su (2021), there are two types of asymptotics for penalized splines: (1) when $\lambda =o(h^{p+q})$, then $h_{e}=h$ and the rates become $O(h^{2r})+o(h^{2p})+O(h^{n^{-1}h^{-1}})$, which are the asymptotic rates for regression splines; (2) when $\lambda^{-1}=o(h^{-2q})$, then $h_{e}=\lambda^{-1∕(2q)}$ and the rates become $O(\lambda )+O(n^{-1}\lambda^{-1∕(2q)})$, the asymptotic rates for smoothing splines. Finally, both types of asymptotics could lead to the best rate, $n^{-2p∕(2p+1)}$, which is the optimal rate for estimating univariate smooth functions (Stone, 1982).

### 3.3 ∣ Asymptotic properties of covariance function estimator

To simplify the theoretic analysis, we shall assume that the mean function μ(t) is known and the auxiliary variables in Section 2.3 are accordingly constructed.

**Assumption 7.** (a) ${\text{sup}}_{t\in 𝒯}E{\left\{\mu^{*}(t,Z_{i})-\mu (t)\right\}}^{4}<\infty$; (b) $\sup_{t\in 𝒯}E{\left\{X_{i}(t)\right\}}^{4}<\infty$; (c) $\sup_{t\in 𝒯}E{\left\{\epsilon_{i}(t)\right\}}^{4}<\infty$.

**Assumption 8.** Let

$$\rho_{3}(s,t_{1},t_{2})\;\;=E\{\Lambda_{0}^{\left(1\right)}(s\;\wedge \;C_{1})\Lambda_{0}^{\left(1\right)}(t_{1}\;\wedge \;C_{1})\Lambda_{0}^{\left(1\right)}(t_{2}\;\wedge \;C_{1})∕\Lambda_{0}^{3}(C_{1})\}$$

and

$$\rho_{4}(s_{1},s_{2},t_{1},t_{2})\;\;=E\{\Lambda_{0}^{\left(1\right)}(s_{1}\;\wedge \;C_{1})\Lambda_{0}^{\left(1\right)}(s_{2}\;\wedge \;C_{1})\Lambda_{0}^{\left(1\right)}\;\;\times \left(t_{1}\;\wedge \;C_{1})\Lambda_{0}^{\left(1\right)}(t_{2}\;\wedge \;C_{1}\right)∕\Lambda_{0}^{4}(C_{1})\}.$$

Both $\rho_{3}$ and $\rho_{4}$ are continuous and bounded away from 0 and infinity, over ${𝒯}^{3}$ and ${𝒯}^{4}$, respectively.

**Theorem 2.** Suppose that Assumptions 1-8 hold. If $\sigma \in {𝒞}^{p}(𝒯)$ with q≤p∧r, then

$$E\left[\int \int {\left\{\hat{\sigma }(s,t)-\sigma (s,t)\right\}}^{2}\;ds\;dt\;|\underline{m},\underline{C}\right]\;\;=O_{P}(h^{2r})+o_{P}(h^{2p})+O_{P}\left(\lambda^{2}h_{e}^{-2q}\right)+O_{P}(n^{-1}h_{e}^{-2}).$$

The proof of Theorem 2 is given in Section B of the Supporting Information. The derived $L_{2}$ rate in Theorem 2 can be shown to achieve the best rate in the literature (Zhang and Wang, 2016; Xiao, 2020), $n^{-2p∕(2p+2)}$, which is the optimal rate for estimating bivariate smooth functions (Stone, 1982).

## 4 ∣ SIMULATION STUDY

### 4.1 ∣ Simulation settings

In this section, we conduct simulation studies to evaluate the performance of the proposed method, that is, penalized splines with SUBJ weights, and compare it with that of penalized splines with OBS weights. In the following simulations, the maximum follow-up time is τ=1 and we consider three censoring time distributions: (1) $C_{i}=1$, which corresponds to no censoring; (2) $C_{i}$ is sampled from a mixture distribution of Uniform(0.2, 1) and a point mass at 1, with mixing factors of 0.8 and 0.2, respectively; and (3) $C_{i}∼\text{Uniform}(0,1)$. For the second generating distribution for the censoring times, which we call mixed censoring, Assumptions 4 and 8 still hold and hence our theoretical results will still hold; see Section C of the Supporting Information for a proof. The third generating distribution, which we call uniform censoring, serves for the purpose of a sensitivity analysis. The frailty variable is chosen as $Z_{i}=(1-\rho )+\rho Z_{i}^{*}$, where $Z_{i}^{*}$ is sampled from a gamma distribution or lognormal distribution, both with mean 1 and variance $\sigma_{Z^{*}}^{2}=0.5$ or $\sigma_{Z^{*}}^{2}=1$, and ρ=0.5 or 0.85, corresponding to the informative observation process settings, and ρ=0, corresponding to the noninformative observation process settings. The observation process follows a Poisson process with the intensity function $10Z_{i}$. Given $Z_{i}$ and $C_{i}$, the number of observations $m_{i}$ of subject i then follows a Poisson distribution with the mean $10Z_{i}C_{i}$.

The conditional mean function is $\mu^{*}(t,Z_{i})=Z_{i}(t^{2}+2∕3)$, implying a marginal mean function of $\mu (t)=t^{2}+2∕3$. The covariance function of the random functions $X_{i}$ is chosen as $\sigma_{X}(s,t)=0.05\;\exp(s^{2})\times 0.05\;\exp(t^{2})\times (1-|s-t|)$. The observed values $Y_{i}(T_{ij})$ are then generated from (1), where $\epsilon_{i}(T_{ij})∼𝒩(0,\sigma_{\epsilon }^{2})$ and $\sigma_{\epsilon }^{2}$ is set to be 0.5 in the informative observation process settings, and 1 in the noninformative observation process settings. The number of subjects is set to *n* = 150 or *n* = 300.

We replicate each simulation setting 200 times. For univariate penalized splines, we use 10 cubic B-spline bases with equally spaced knots in the unit interval and for the tensor-product penalized splines, we use 10 marginal cubic B-splines with equally spaced knots in both dimensions. The smoothing parameters are selected using generalized cross-validation via a modified version of the function *fpca.sparse* in the R package *face*, which performs the fast covariance estimation (FACE) method for sparse functional data (Xiao et al., 2018).

### 4.2 ∣ Simulation results

Define the integrated squared error as either $\int {\left\{\hat{\mu }(t)-\mu (t)\right\}}^{2}dt$ or $\int \int {\left\{\hat{\sigma }(s,t)-\sigma (s,t)\right\}}^{2}ds\;dt$ where $\hat{\mu }(t)$ and $\hat{\sigma }(s,t)$ are estimates of μ(t) and σ(s,t). For each simulation setting, we calculate the median and interquartile range of the integrated squared error of the estimates using the OBS and SUBJ choices of weights.

**FIGURE 2:**
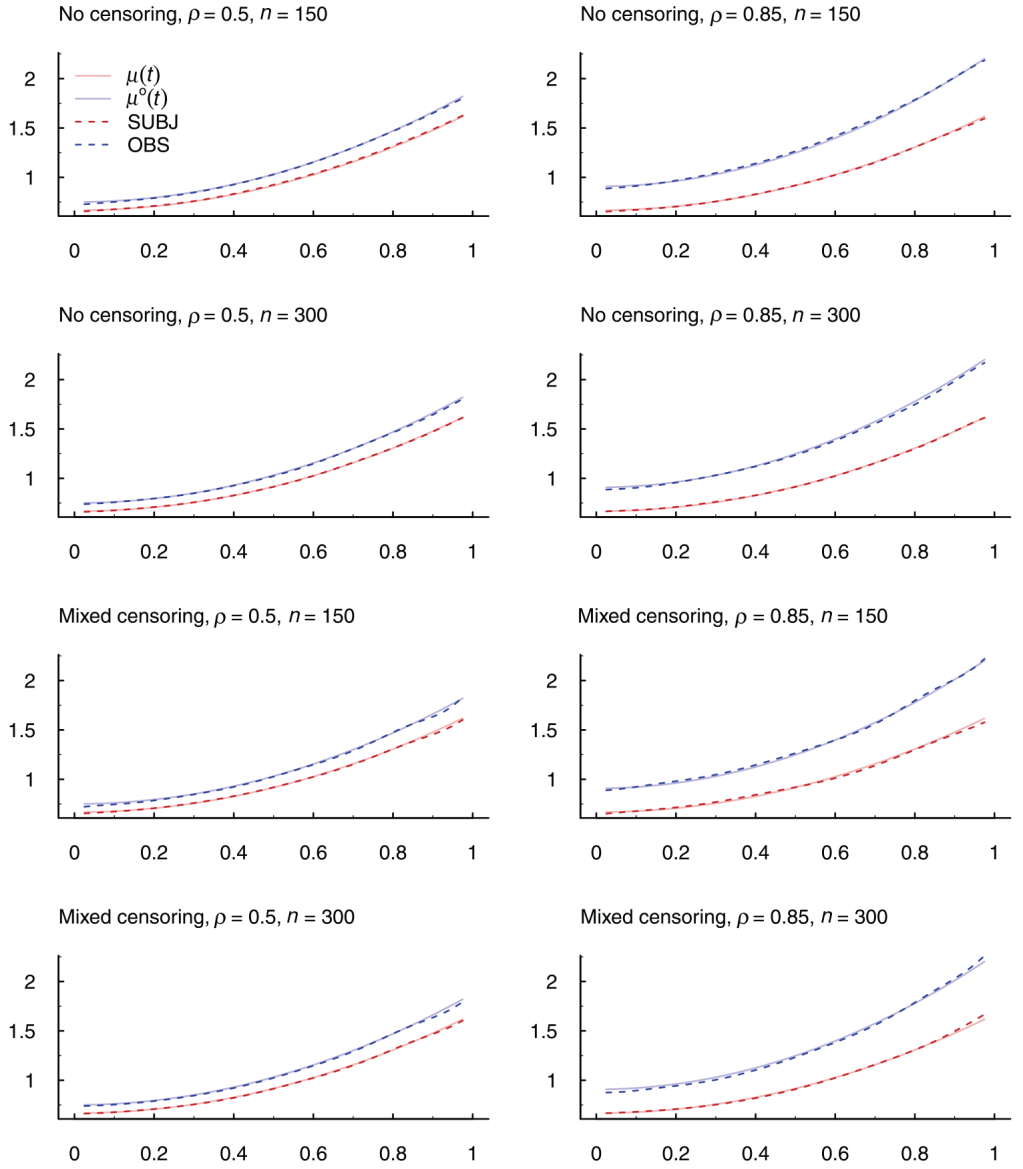
Average of 200 estimates of the mean function using penalized splines with both OBS and SUBJ weights for simulation settings in which the frailty variable $Z_{i}^{*}$ follows a gamma distribution with variance $\sigma_{Z^{*}}^{2}=0.5$. The true mean function is $\mu (t)=t^{2}+2∕3$ and $\mu^{o}(t)$ is the formula in (4) associated with OBS weights, specifically $\mu^{o}(t)=(t^{2}+2∕3)(1+\rho^{2}\sigma_{Z^{*}}^{2})$.

Figure 2 plots the averages of the estimated mean functions from penalized splines with either OBS or SUBJ weights for a few simulation settings with gamma frailty variables, for no censoring and mixed censoring times. In these plots, $\sigma_{Z^{*}}^{2}=0.5$. A nonzero value of ρ in these plots indicate informative observation times. We first observe from the plots that penalized splines with OBS weights converge to $\mu^{o}(t)=(t^{2}+2∕3)(1+\rho^{2}\sigma_{Z^{*}}^{2})$, the derived expression in (4) for OBS weights, which differs from the true $\mu (t)=t^{2}+2∕3$ and the difference becomes more pronounced as ρ increases. Next we see that penalized splines with SUBJ weights converge to the true mean function as desired, irrespective of the values of ρ. Thus, the plots demonstrate the inconsistent estimation of the mean function by penalized splines with OBS weights and the desired model estimation by penalized splines with SUBJ weights. The plots for uniform censoring and lognormal frailty variables are similar and are presented in Web Appendix D.

Table 1 gives the median and interquantile range of integrated squared error for estimating the mean function using both methods in all simulation settings with informative observation times. The numerical results show that penalized splines with SUBJ weights have much smaller median integrated squared error for estimating the mean function, in agreement with the above plots. In addition, the estimators based on the SUBJ weights have much smaller variation in terms of interquartile range in the presence of information observation times. While there is some additional variability in the estimates at the larger time points in the mixed censoring settings compared to the no censoring settings, censoring seems to have a less significant effect on the estimates. Similar simulation results for the uniform censoring setting are found and given in Web Appendix D.

**FIGURE 3:**
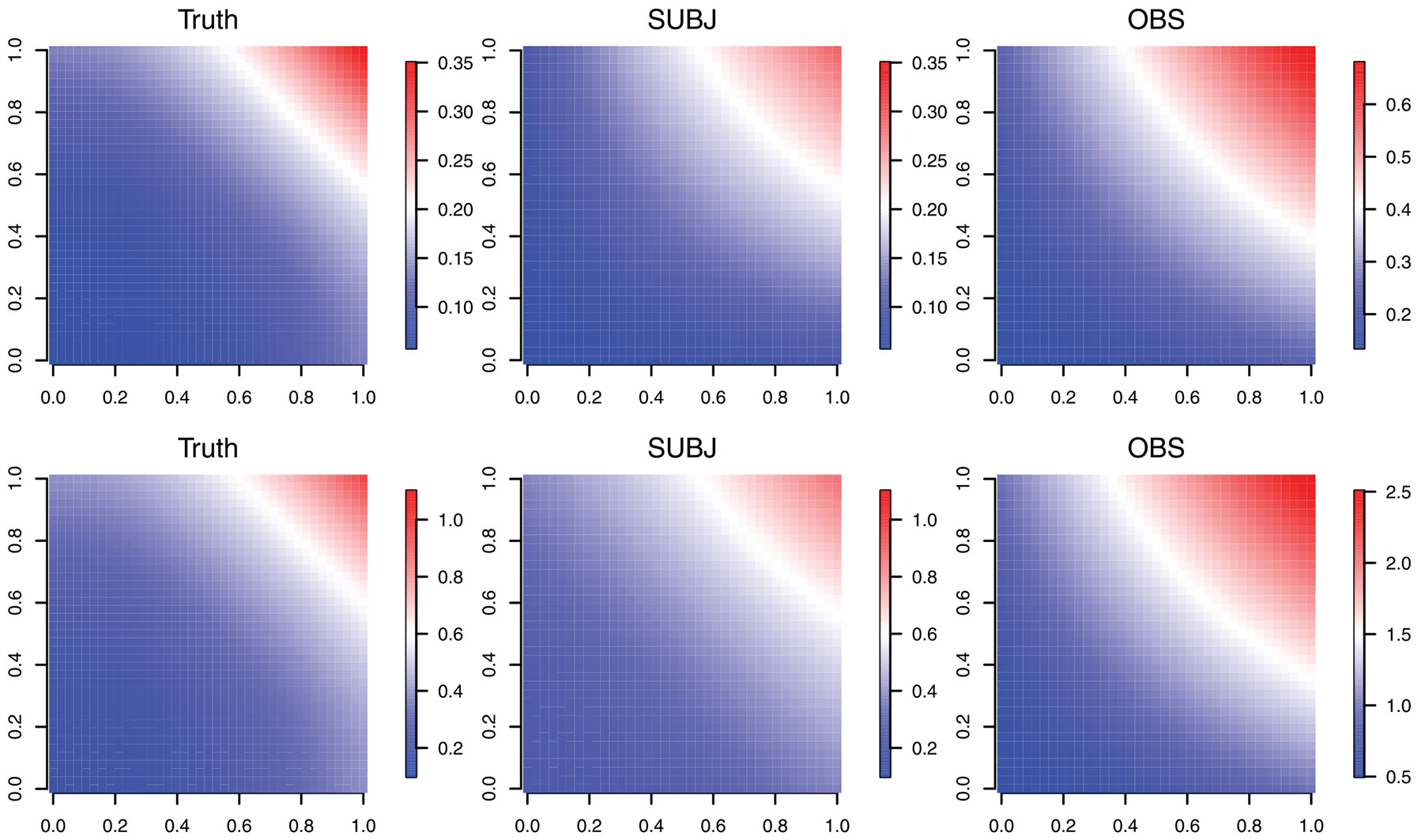
True covariance function and corresponding estimates using penalized splines with both OBS and SUBJ weights for two simulated data sets in which the frailty variable $Z_{i}^{*}$ follows a gamma distribution with variance $\sigma_{Z^{*}}^{2}=0.5$, with ρ=0.5 (top row) and ρ=0.85 (bottom row).

The numerical results for the covariance function estimation under the informative observation times settings are summarized in Table 2, and the corresponding numerical results under the noninformative observation times settings are included in Section D of the Supporting Information. The estimate using the SUBJ weights clearly outperforms the estimate using the OBS weights in both cases. Similar to the results for the mean function estimation, the estimators based on the SUBJ weights have much smaller integrated squared error and variation than those based on the OBS weights under all the settings with information observation times. For illustration, Figure 3 presents the estimated covariance function from both methods along with the true covariance function from two simulated data sets.

In simulation settings with noninformative observations, that is, ρ=0, we find no substantial difference in the integrated squared errors for estimating the mean and covariance functions using either the SUBJ or OBS weights. Indeed, penalized splines with both types of weights perform similarly and both estimate accurately the mean function. The numerical results are given in Web Appendix D.

Based on the above empirical results, we can recommend the use of the SUBJ weights for any longitudinal data with or without informative observations, without concern for suboptimal estimation in the case that the observation process is noninformative.

## 5 ∣ DATA APPLICATION

In this section, we apply the discussed methods to the Parkinson’s disease symptom severity data set described in Section 1. Recall that in this data set, there was evidence of an informative observation process in which the average severity score of subjects with more than 10 observations is less than that of subjects with less than or equal to 10 observations. The intuition behind the selection of weights is clearly illustrated by this example. When weighting each observation equally, the estimates are more heavily influenced toward subjects with more observations. Thus, the mean function is likely to be underestimated. Similarly, the estimate of the covariance function is likely to be biased. Also recall that the time at which a patient entered the study is unrelated to the time since their initial diagnosis, there is no instance of dropout, and we only consider data observed less than 20 years following a patient’s diagnosis. There is therefore no informative truncation or censoring present in the data. Therefore, it seems appropriate to use the proposed functional method with SUBJ weights.

**FIGURE 4:**
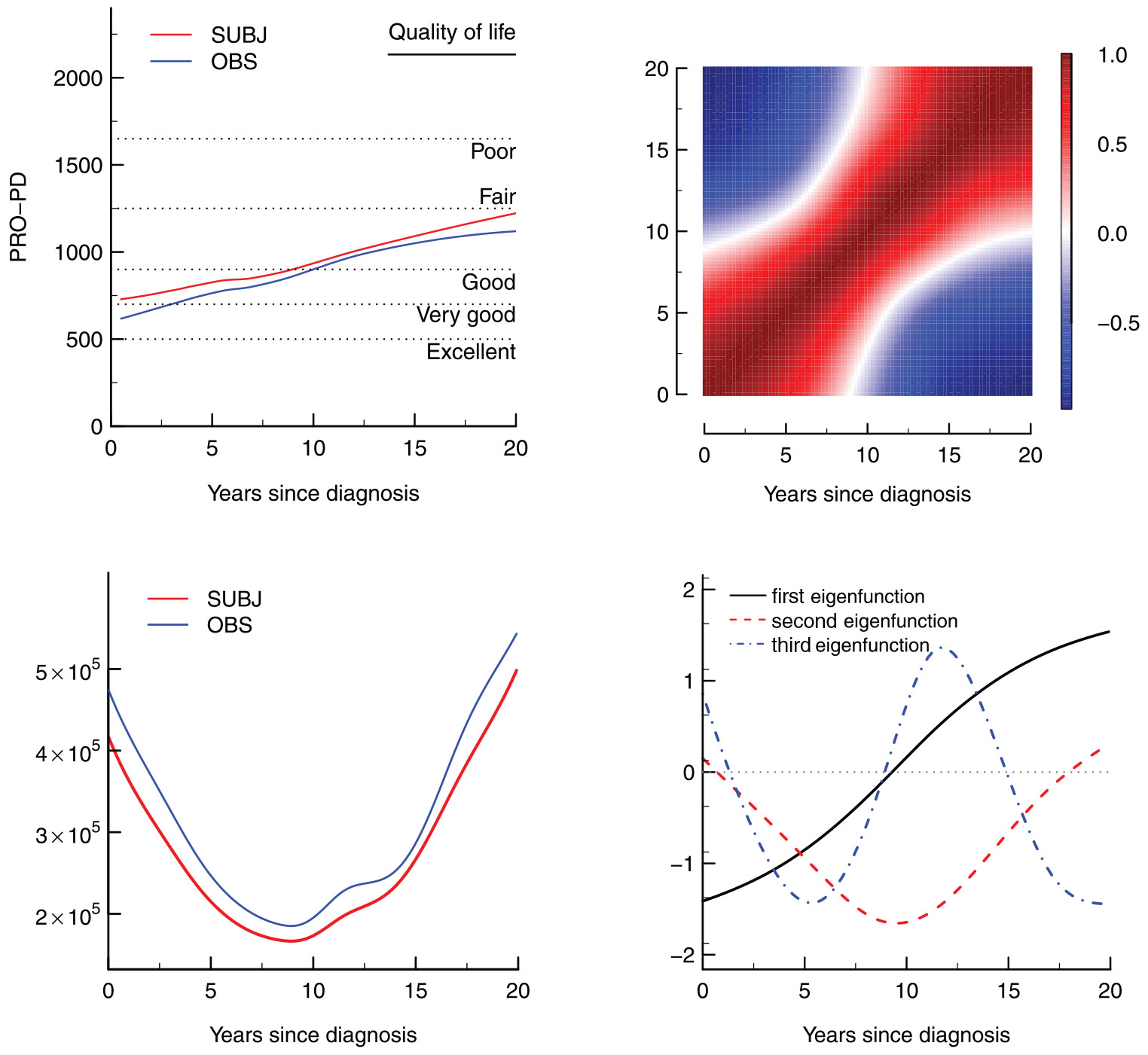
(Top left) Mean function estimates using both the SUBJ and OBS weights compared to average PRO-PD by reported quality of life. (Top right) Estimated correlation function using the SUBJ weights. (Bottom left) Estimated variance function using both the SUBJ and OBS weights. (Bottom right) First three estimated eigenfunctions using the SUBJ weights.

The trajectories are plotted in Figure 4 along with the mean function estimates of the penalized spline estimator with the OBS and SUBJ weights. As expected, the mean estimate when using SUBJ weights is greater than the estimate when using OBS weights over the whole time domain. By the estimate from penalized splines with SUBJ weights, we see a monotonic increase in severity over the first 20 years after diagnosis, which agrees with existing research (Group, 2004; Venuto et al., 2016; Holden et al., 2018).

The estimated correlation and variance functions of the penalized spline estimator with SUBJ weights are displayed in Figure 4. It is interesting to see that the variance function takes its smallest value around 8 years after diagnosis and is greatest at the maximal follow-up time. As a comparison, the estimated variance function with OBS weights is also plotted and has uniformly higher values. We also consider the spectral decomposition of the estimated covariance function. The first three eigenfunctions are shown in Figure 4. These first three eigenfunctions account for 60%, 30%, and 6% of the total variation, respectively. The first eigenfunction corresponds to a contrast between early and late time after diagnosis. The second accounts for deviation from the mean trajectory around 8 years after diagnosis. The estimated correlation function and associated eigenfunctions using OBS weightings are displayed in Section E of the Supporting Information and very similar to the above results using SUBJ weights.

## 6 ∣ DISCUSSION

In this paper, we studied some commonly used functional data methods for analysis of longitudinal data with informative observation times and identified a method with a proper subject-specific weighting scheme that can achieve the consistent estimation of the mean and covariance functions under a class of shared random effect models. The suggested method can be easily implemented using existing software. The theoretical properties of the estimator were studied under the setting with the informative observation process, and special care must be taken to handle the distribution of observation times for establishing the convergence rate results.

In our current work, no covariates are included in the model. But the proposed functional data method can be easily extended to accommodate covariates. In addition, censoring times are assumed to be independent of longitudinal outcomes and observation times, which we think is reasonable for our data application. It is certainly of interest to extend the proposed method to the setting with the informative observation process and informative dropout. In such a setting, the shared random effects model can be extended to account for dependence between censoring times and longitudinal outcomes and observation times. Then, the nonparametric approach in Wang et al. (2001) can be used to estimate the baseline cumulative intensity function. Note that the derivations that lead to Equation (4) remain valid for informative censoring. Therefore, Equation (4) may be used for selecting an appropriate set of weights in aggregating the observations. Such a research direction merits further investigation.

## Supplementary Material

Supplemental material

Web Appendices, Tables, and Figures referenced in Sections 4 and 5, and R codes are available with this paper at the Biometrics website on Wiley Online Library. The R codes used in the simulation study are also available online at https://github.com/clbwvr/fda_io.

## Figures and Tables

**TABLE 1:** Median (interquartile range) of integrated squared error for estimating the mean function using penalized splines with both OBS and SUBJ weights

			Gamma			
			No censoring		Mixed censoring	
ρ	n	$\sigma_{Z^{*}}^{2}$	OBS	SUBJ	OBS	SUBJ
0.50	150	0.5	0.011 (0.017)	0.001 (0.002)	0.022 (0.021)	0.002 (0.002)
		1.0	0.059 (0.060)	0.002 (0.002)	0.068 (0.039)	0.004 (0.004)
	300	0.5	0.016 (0.008)	0.000 (0.000)	0.011 (0.007)	0.001 (0.002)
		1.0	0.069 (0.037)	0.001 (0.001)	0.073 (0.042)	0.003 (0.003)
0.85	150	0.5	0.171 (0.109)	0.003 (0.003)	0.102 (0.116)	0.007 (0.010)
		1.0	0.557 (0.467)	0.007 (0.009)	0.409 (0.343)	0.016 (0.038)
	300	0.5	0.141 (0.056)	0.001 (0.002)	0.102 (0.057)	0.005 (0.008)
		1.0	0.596 (0.184)	0.003 (0.003)	0.593 (0.297)	0.011 (0.014)
			Lognormal			
			No censoring		Mixed censoring	
ρ	n	$\sigma_{Z^{*}}^{2}$	OBS	SUBJ	OBS	SUBJ
0.50	150	0.5	0.016 (0.024)	0.001 (0.002)	0.012 (0.027)	0.002 (0.003)
		1.0	0.073 (0.120)	0.001 (0.002)	0.075 (0.081)	0.005 (0.005)
	300	0.5	0.017 (0.014)	0.000 (0.000)	0.017 (0.017)	0.001 (0.001)
		1.0	0.048 (0.061)	0.001 (0.001)	0.054 (0.078)	0.002 (0.003)
0.85	150	0.5	0.141 (0.048)	0.003 (0.004)	0.098 (0.137)	0.009 (0.008)
		1.0	0.477 (0.344)	0.006 (0.009)	0.297 (0.684)	0.014 (0.012)
	300	0.5	0.102 (0.052)	0.001 (0.002)	0.107 (0.091)	0.005 (0.005)
		1.0	0.579 (0.348)	0.003 (0.003)	0.375 (0.283)	0.005 (0.009)

**TABLE 2:** Median (interquartile range) of integrated squared error for estimating the covariance function using penalized splines with both OBS and SUBJ weights

			Gamma			
			No censoring		Mixed censoring	
ρ	n	$\sigma_{Z^{*}}^{2}$	OBS	SUBJ	OBS	SUBJ
0.50	150	0.5	0.018 (0.015)	0.000 (0.001)	0.011 (0.066)	0.001 (0.002)
		1.0	0.224 (0.653)	0.004 (0.009)	0.242 (1.141)	0.008 (0.014)
	300	0.5	0.009 (0.010)	0.000 (0.000)	0.006 (0.041)	0.001 (0.001)
		1.0	0.220 (0.472)	0.003 (0.004)	0.211 (0.789)	0.005 (0.005)
0.85	150	0.5	0.324 (0.581)	0.008 (0.014)	0.033 (0.226)	0.011 (0.018)
		1.0	5.359 (21.07)	0.043 (0.157)	5.568 (22.08)	0.077 (0.049)
	300	0.5	0.235 (0.399)	0.003 (0.003)	0.068 (0.577)	0.006 (0.013)
		1.0	3.022 (15.30)	0.045 (0.060)	2.556 (5.849)	0.054 (0.059)
			Lognormal			
			No censoring		Mixed censoring	
ρ	n	$\sigma_{Z^{*}}^{2}$	OBS	SUBJ	OBS	SUBJ
0.50	150	0.5	0.032 (0.109)	0.001 (0.002)	0.011 (0.160)	0.002 (0.004)
		1.0	2.885 (7.116)	0.011 (0.043)	0.341 (1.786)	0.012 (0.028)
	300	0.5	0.093 (0.113)	0.001 (0.002)	0.043 (0.088)	0.001 (0.002)
		1.0	0.614 (2.761)	0.005 (0.006)	0.747 (12.21)	0.010 (0.021)
0.85	150	0.5	0.691 (1.745)	0.007 (0.011)	0.652 (5.084)	0.031 (0.042)
		1.0	12.37 (74.19)	0.034 (0.106)	9.179 (96.96)	0.176 (0.238)
	300	0.5	0.550 (1.728)	0.004 (0.005)	0.562 (2.247)	0.009 (0.021)
		1.0	32.76 (93.44)	0.076 (0.133)	10.48 (129.7)	0.060 (0.078)

## Data Availability

The data that support the findings in this paper are available on request from the corresponding author. The data are not publicly available due to privacy or ethical restrictions.
