# Supplementary material for: Functional data analysis for longitudinal data with informative observation times
Source: Biometrics. Author manuscript; Available in PMC 2026 May 8. (PMC13154409; doi:10.1111/biom.13646)
Supplement: Supplemental material [file NIHMS2166927-supplement-Supplemental_material.pdf]

BIOMETRICS 000, 000–000 DOI: 000
000 0000

Supporting Information for Functional Data Analysis for Longitudinal Data with Informative Observation Times

by Caleb Weaver, Luo Xiao, and Wenbin Lu

This paper has been submitted for consideration for publication in *Biometrics*

Web Appendix A. Technical Lemmas for the Proof of Theorem 1

First, we define some notation. Let $\tau_1 = \sum_{i=1}^n w_i^2 m_i$ and $\tau_2 = w_i^2 m_i(m_i - 1)$. Define $Q_{ni}(t) = m_i^{-1} N_i(t)$, $Q_n(t) = \sum_{i=1}^n w_i m_i Q_{ni}(t)$ and $\widetilde{Q}_n(t) = \tau_1^{-1} \sum_{i=1}^n w_i^2 m_i Q_{ni}(t)$. Both $Q_n(t)$ and $\widetilde{Q}_n(t)$ are empirical cumulative distribution functions (CDF) and they shall be proved to converge to proper CDFs. Moreover, let $R_n(s,t) = \tau_2^{-1} \sum_{i=1}^n \sum_{j_1 \neq j_2} w_i^2 I(T_{ij_1} \leqslant s) I(T_{ij_2} \leqslant t)$, which is a bivariate empirical CDF.

In Lemmas 1-3 below, suppose that Assumptions 1-6 hold.

Lemma 1: *Define*

$$Q(t) = \mathbb{E}\{\Lambda_0(t \wedge C_1)/\Lambda_0(C_1)\}$$

and

$$R(s,t) = \mathbb{E}\{\Lambda_0(t \wedge C_1)\Lambda_0(s \wedge C_1)/\Lambda_0^2(C_1)\}.$$

If $\log n/n = o(h^4)$*, the following results hold almost surely:*

(i) $\|Q_n - Q\| = o(h)$.

(ii) $\|\widetilde{Q}_n - Q\| = o(h)$.

(iii) Let $U(s,t) = R_n(s,t) - R(s,t)$*. Then,* $\|U\| = o(h^2)$.

Proof of Lemma 1. We first consider (i) and have

$$\mathbb{E}\{\mathrm{d}N_i(t) \mid m_i, C_i\} = I(C_i \geqslant t) m_i \frac{\mathrm{d}\Lambda_0(t)}{\Lambda_0(C_i)}.$$

Hence,

$$\begin{aligned}\mathbb{E}\{Q_{ni}(t) \mid m_i, C_i\} =& \mathbb{E}\left\{\frac{1}{m_i}\int_0^t \mathrm{d}N_i(s) \mid m_i, C_i\right\}\\ =& \int_0^t I(C_i \geqslant s)\frac{\mathrm{d}\Lambda_0(s)}{\Lambda_0(C_i)}\\ =& \frac{\Lambda_0(t \wedge C_i)}{\Lambda_0(C_i)}.\end{aligned}$$

Let

$$F_n(t) = n^{-1}\sum_{i=1}^{n}\frac{\Lambda_0(t\wedge C_i)}{\Lambda_0(C_i)}.$$

Define $S_{ni}(t) = Q_{ni}(t) - \Lambda_0(t\wedge C_i)/\Lambda_0(C_i)$ and $S_n(t) = Q_n(t) - F_n(t)$. As $w_i = (nm_i)^{-1}$, $S_n(t) = n^{-1}\sum_{i=1}^{n} S_{ni}(t)$. We have $\mathbb{E}[\{S_{ni}(t)\}^2 \mid m_i, C_i] \leqslant m_i^{-1}$ and thus $\mathbb{E}[\{S_n(t)\}^2 \mid m_i, C_i, 1\leqslant i\leqslant n] \leqslant n^{-1}$ for any t. Then by using the same technique as in the proof of Lemma A.7 in Xiao (2020), $\|Q_n - F_n\| = o(h)$ almost surely as we assume $\log n/n = o(h^4)$. Note that $\Lambda_0(t\wedge C_i)/\Lambda_0(C_i)$ is a non-decreasing function of t and is bounded by 1. Then similarly, it can be shown that $\|F_n - Q\| = o(h)$ almost surely.

Next for (ii), let

$$\widetilde{F}_n(t) = \tau_1^{-1}\sum_{i=1}^{n} w_i^2 m_i\frac{\Lambda_0(t\wedge C_i)}{\Lambda_0(C_i)}.$$

Then, we derive that $\|\widetilde{Q}_n - \widetilde{F}_n\| = o(h)$ as long as Lemma 2 (i) and (ii) hold almost surely. Similarly, we could show that $\|\widetilde{F}_n - Q\| = o(h)$ almost surely.

Finally, let

$$F_n(s,t) = \tau_2^{-1}\sum_{i=1}^{n} w_i^2 m_i(m_i-1)\frac{\Lambda_0(s\wedge C_i)\Lambda_0(t\wedge C_i)}{\Lambda_0^2(C_i)}.$$

Then, we derive that $\|R_n - F_n\| = o(h^2)$ almost surely if Lemma 2 (iii) and (iv) hold almost surely. Similarly, we could show that $\|F_n - R\| = o(h^2)$ almost surely.

LEMMA 2: *If $\log n/n = o(h^4)$, the following results hold almost surely:*

(i) $\tau_1^{-2}\left(\sum_{i=1}^{n} m_i w_i^4\right) = o(h^2/\log n)$.

(ii) $\tau_1^{-1}\max_i m_i w_i^2 = o(h/\log n)$.

(iii) $\tau_2^{-2}\left\{\sum_{i=1}^{n} m_i(m_i-1)(4m_i-6)w_i^4\right\} = o(h^4/\log n)$.

(iv) $\tau_2^{-1}\max_i m_i(m_i-1)w_i^2 = o(h^2/\log n)$.

Proof of Lemma 2. First consider (i) and (ii). We have $\tau_1 = n^{-2}\sum_i m_i^{-1}$. For (iii), we

have

$$\tau_1^{-2}\left(\sum_i m_i w_i^4\right) \leqslant \left(\sum_i m_i^{-1}\right)^{-1} = O(n^{-1})$$

almost surely by Lemma 3 and then (i) holds.

For (ii),

$$\tau_1^{-1}\max_i m_i w_i^2 \leqslant \left(\sum_i m_i^{-1}\right)^{-1} = O(n^{-1}).$$

Hence, (ii) holds.

Next consider (iii) and (iv). We have

$$\tau_2 = n^{-2}\sum_i \left(1 - m_i^{-1}\right).$$

For (iii),

$$\tau_2^{-2}\left\{\sum_{i=1}^n m_i(m_i-1)(4m_i-6)w_i^4\right\} \leqslant \frac{4\sum_i m_i^{-1}}{\left\{\sum_i \left(1-m_i^{-1}\right)\right\}^2}.$$

For (iv),

$$\tau_2^{-1}\max_i m_i(m_i-1)w_i^2 \leqslant \left\{\sum_i \left(1-m_i^{-1}\right)\right\}^{-1}.$$

Thus, by Lemma 3 and the assumption that $\log n/n = o(h^4)$, both (iii) and (iv) hold.

Lemma 3: *There exist constants* $0 < c_1 < c_2 < 1$ *such that*

$$c_1 < n^{-1}\sum_i I(m_i \neq 0)m_i^{-1} < c_2, \qquad \textit{almost surely.}$$

Proof of Lemma 3. Let $a_i = I(m_i \geqslant 0)m_i^{-1}$, then marginally $a_i\,(1 \leqslant i \leqslant n)$ are independent and identically distributed. By the strong law of large numbers, $n^{-1}\sum_i I(m_i \neq 0)m_i^{-1}$ converges almost surely to $\mathbb{E}(a_1)$, which is bounded by $c_2 = (1+e^{-2})/2$. Indeed, by Lemma 4, $\mathbb{E}(a_1) = \mathbb{E}\{\mathbb{E}(a_1 \mid Z_1, C_1)\} \leqslant \mathbb{E}(c_2) = c_2$. The proof is complete if $\mathbb{E}(a_1) > c_1$ for some constant $c_1 > 0$. First, we have $\mathbb{E}(a_1 \mid Z_1, C_1) > \lambda_1 e^{-\lambda_1}$, where $\lambda_1 = c_0 Z_1 \Lambda_0(C_1)$. Thus,

$$\mathbb{E}(a_1 \mid Z_1) \geqslant \mathbb{E}\left\{c_0 Z_1 \Lambda_0(C_1) e^{-c_0 Z_1 \Lambda_0(C_1)} \mid Z_1\right\} \geqslant c_0 \mathbb{E}\{\Lambda_0(C_1)\} Z_1 e^{-c_0 Z_1 \Lambda_0(1)}$$

and

$$\mathbb{E}(a_1) \geqslant c_0 \mathbb{E}\{\Lambda_0(C_1)\}\mathbb{E}\left\{Z_1 e^{-c_0 Z_1 \Lambda_0(1)}\right\}.$$

Let c_1 be the right hand side of the above inequality. By Assumptions 1 and 2, $c_1 > 0$. The proof is now complete.

LEMMA 4: *If m has a Poisson distribution with mean $\lambda \geqslant 0$. Then*

$$\mathbb{E}\{I(m \neq 0)m^{-1}\} \leqslant \left(1 + e^{-2}\right)/2.$$

Proof of Lemma 4. We derive that

$$\begin{aligned}
\mathbb{E}\{I(m \neq 0)m^{-1}\} &= \sum_{k=1}^{\infty} \frac{1}{k} \frac{e^{-\lambda}\lambda^k}{k!} \\
&\leqslant \frac{1}{2} e^{-\lambda}\lambda + \frac{1}{2} \sum_{k=1}^{\infty} \frac{e^{-\lambda}\lambda^k}{k!} \\
&\leqslant \frac{1}{2} e^{-\lambda}\lambda + \frac{1}{2}(1 - e^{-\lambda}) \\
&\leqslant \frac{1}{2}\{1 + (\lambda - 1)e^{-\lambda}\}.
\end{aligned}$$

Let $f(\lambda) = (\lambda - 1)e^{-\lambda}$. It is easy to check that f has maximum at $\lambda = 2$. Thus, for any $\lambda \geqslant 0$,

$$\mathbb{E}\{I(m \neq 0)m^{-1}\} \leqslant 2^{-1}\{1 + f(2)\} = 2^{-1}(1 + e^{-2}).$$

Web Appendix B. Proof of Theorem 2

First, we define some additional notation. The Euclidean norm is $\|\cdot\|_2$ and the operator norm is $\|\cdot\|_{op}$. For a matrix $A = (a_{ij})$, $\|A\|_{\max} = \max_{i,j} |a_{ij}|$ and $\|A\|_\infty = \max_i \sum_j |a_{ij}|$.

Let a and b be a sequence of scalars that depend on n. The notation $a \preceq b$ denotes that there exists an absolute constant $c > 0$ such that $a \leqslant cb$ for sufficiently large n. And $a \simeq b$ means that $a \preceq b$ and $b \preceq a$, i.e., a and b are rate-wise equivalent. For two square matrices A and B, $A \leqslant B$ denotes that $B - A$ is elementwise nonnegative, $A \preceq B$ means that there exists an absolute constant $c > 0$ such that $A \leqslant cB$ for sufficiently large n, and $A \simeq B$ means that $A \preceq B$ and also $B \preceq A$.

Also to simplify notation, in the rest of the proof, unless otherwise noted, the expectation and variance are conditional on $\underline{m}$ and $\underline{C}$.

First, by symmetry of the auxiliary variables, i.e., $\widetilde{\sigma}_{ij_1j_2} = \widetilde{\sigma}_{ij_2j_1}$, it can be shown that $\widehat{\Theta}$, the solution to (5), is also the minimizer of the following optimization problem,

$$\widehat{\Theta} = \arg\min_{\Theta} \sum_{i=1}^{n} \left[v_i \sum_{1 \leqslant j_1 \neq j_2 \leqslant m_i} \{\widetilde{\sigma}_{ij_1j_2} - H(T_{ij_1}, T_{ij_2})\}^2 \right] + \boldsymbol{\theta}_\sigma' P_\sigma \boldsymbol{\theta}_\sigma, \tag{S.1}$$

where $P_\sigma = 2^{-1}\lambda K^{-1}(I_K \otimes P + P \otimes I_K)$. We shall use the above formulation to derive an explicit expression of $\widehat{\sigma}(s,t)$. To simplify notation, we shall drop the subscript in λ_σ in the following derivation.

Let $e_{ij} = Y_{ij} - \mu(T_{ij})$ and $\widetilde{\sigma}_{ij_1j_2} = e_{ij_1}e_{ij_2}$. Then let $\Sigma_i = \{\sigma(T_{ij_1}, T_{ij_2})\}_{1 \leqslant j_1, j_2 \leqslant m_i} \in \mathbb{R}^{m_i \times m_i}$ and $\boldsymbol{\sigma}_i = \text{vec}^*(\Sigma_i)$, where vec* is a matrix operator that is the same as vec$(\cdot)$ except it excludes the diagonal elements of a square matrix. Similarly, let $\widehat{\Sigma}_i = (\widetilde{\sigma}_{ij_1j_2})_{1 \leqslant j_1, j_2 \leqslant m_i}$ and $\widehat{\boldsymbol{\sigma}}_i = \text{vec}^*(\widehat{\Sigma}_i)$. Also let $B_i = [B(T_{i1}), \ldots, B(T_{im_i})]' \in \mathbb{R}^{m_i \times K}$ and A_i be the sub-matrix of $B_i \otimes B_i$ that excludes the rows corresponding to the same T_{ij}. Finally, let $V_i = v_i I_{m_i(m_i-1)}$. Then, the objective function in equation (S.1) becomes

$$\sum_{i=1}^{n} (\widetilde{\boldsymbol{\sigma}}_i - A_i\boldsymbol{\theta}_\sigma)^\mathsf{T} V_i (\widetilde{\boldsymbol{\sigma}}_i - A_i\boldsymbol{\theta}_\sigma) + \boldsymbol{\theta}_\sigma' P_\sigma \boldsymbol{\theta}_\sigma.$$

Now let $\boldsymbol{\sigma} = (\boldsymbol{\sigma}_1^\mathsf{T}, \ldots, \boldsymbol{\sigma}_n^\mathsf{T})^\mathsf{T}$, $\widetilde{\boldsymbol{\sigma}} = (\widetilde{\boldsymbol{\sigma}}_1^\mathsf{T}, \ldots, \widetilde{\boldsymbol{\sigma}}_n^\mathsf{T})^\mathsf{T}$, $V = \text{blockdiag}(V_1, \ldots, V_n)$, and $A = [A_1^\mathsf{T}, \ldots, A_n^\mathsf{T}]^\mathsf{T}$. Finally, let $G_{\sigma,n} = A^\mathsf{T} VA$ and $H_{\sigma,n} = G_{\sigma,n} + P_\sigma$. Then we derive

$$\widehat{\sigma}(s,t) = B^\mathsf{T}(s,t)\widehat{\boldsymbol{\theta}}_\sigma = B^\mathsf{T}(s,t) H_{\sigma,n}^{-1}(A^\mathsf{T} V\widetilde{\boldsymbol{\sigma}}).$$

We now consider $\|\mathbb{E}\widehat{\sigma} - \sigma\|$ and derive that

$$\begin{aligned}\mathbb{E}\widehat{\sigma}(s,t) =& B^\mathsf{T}(s,t) H_{\sigma,n}^{-1}(A^\mathsf{T} V\boldsymbol{\sigma}) \\ =& B^\mathsf{T}(s,t) G_{\sigma,n}^{-1}(A^\mathsf{T} V\boldsymbol{\sigma}) - B^\mathsf{T}(s,t) H_{\sigma,n}^{-1} P_\sigma G_{\sigma,n}^{-1}(A^\mathsf{T} V\boldsymbol{\sigma}).\end{aligned}$$

Let η_σ be as in Lemma S.3.1 in the supplement of Xiao (2020) and such that

$$\|\sigma - \eta_\sigma\| = O(h_\sigma^r) + o\left(h_\sigma^p\right). \tag{S.2}$$

Note, $B^{\mathsf{T}}(s,t)G_{\sigma,n}^{-1}(A^{\mathsf{T}}V\boldsymbol{\eta}_\sigma) = \eta_\sigma(s,t)$, where $\boldsymbol{\eta}_\sigma$ is similarly defined as $\boldsymbol{\sigma}$. Thus,

$$\mathbb{E}\widehat{\sigma}(s,t) = \eta_\sigma(s,t) + B^{\mathsf{T}}(s,t)G_{\sigma,n}^{-1}\left\{A^{\mathsf{T}}V(\boldsymbol{\sigma}-\boldsymbol{\eta}_\sigma)\right\} - B^{\mathsf{T}}(s,t)H_{\sigma,n}^{-1}P_\sigma G_{\sigma,n}^{-1}(A^{\mathsf{T}}V\boldsymbol{\sigma}),$$

Let $\boldsymbol{\alpha}_\sigma = A^{\mathsf{T}}V(\boldsymbol{\sigma}-\boldsymbol{\eta})$ and $\boldsymbol{\gamma}_\sigma = G_{\sigma,n}^{-1}(A^{\mathsf{T}}V\boldsymbol{\sigma})$. Then,

$$(\mathbb{E}\widehat{\sigma}-\sigma)(s,t) = (\eta_\sigma-\sigma)(s,t) + B^{\mathsf{T}}(s,t)G_{\sigma,n}^{-1}\boldsymbol{\alpha}_\sigma - B^{\mathsf{T}}(s,t)H_{\sigma,n}^{-1}P_\sigma\boldsymbol{\gamma}_\sigma.$$

It follows that

$$\begin{aligned}&\frac{1}{3}\iint\{\mathbb{E}\widehat{\sigma}(s,t)-\sigma(s,t)\}^2 d_s d_t R(s,t)\\ &\leqslant \iint\{\eta_\sigma(s,t)-\sigma(s,t)\}^2 d_s d_t R(s,t) + \boldsymbol{\alpha}_\sigma^{\mathsf{T}}G_{\sigma,n}^{-1}G_\sigma G_{\sigma,n}^{-1}\boldsymbol{\alpha}_\sigma + \boldsymbol{\gamma}_\sigma' P_\sigma H_{\sigma,n}^{-1}G_\sigma H_{\sigma,n}^{-1}P_\sigma\boldsymbol{\gamma}_\sigma,\end{aligned} \tag{S.3}$$

where $G_\sigma = \iint\{B(s)B^{\mathsf{T}}(s)\}\otimes\{B(t)B^{\mathsf{T}}(t)\}d_s d_t R(s,t)$.

Let $\alpha_{\sigma,k\ell}$ be the (k,ℓ)th element of $\boldsymbol{\alpha}_\sigma$. Then

$$\begin{aligned}\alpha_{\sigma,k\ell} &= \sum_{i=1}^{n}\sum_{1\leqslant j_1\neq j_2\leqslant m_i} v_i B_k(T_{ij_1})B_\ell(T_{ij_2})\left\{\sigma(T_{ij_1},T_{ij_2})-\eta_\sigma(T_{ij_1},T_{ij_2})\right\}\\ &= \iint B_k(s)B_\ell(t)\left\{\sigma(s,t)-\eta_\sigma(s,t)\right\}d_s d_t R_n(s,t).\end{aligned}$$

By Lemma S.3.2 in the supplement of Xiao (2020) and Lemma 1 (*iii*), it can be shown that $\|\boldsymbol{\alpha}_\sigma\|_{\max} = o(h^{p+2})$. Then, by Lemma 6,

$$\boldsymbol{\alpha}_\sigma^{\mathsf{T}}G_{\sigma,n}^{-1}G_\sigma G_{\sigma,n}^{-1}\boldsymbol{\alpha}_\sigma = o(h^p), \text{ a.s.} \tag{S.4}$$

Next by a proof similar to that of (8.15) in Xiao (2019a), we derive that

$$\boldsymbol{\gamma}_\sigma' P_\sigma H_{\sigma,n}^{-1}G_\sigma H_{\sigma,n}^{-1}P_\sigma\boldsymbol{\gamma}_\sigma = O\left\{\left(1\vee\|G_{\sigma,n}^{-1/2}P_\sigma G_{\sigma,n}^{-1/2}\|_{op}\right)\boldsymbol{\gamma}_\sigma' P_\sigma\boldsymbol{\gamma}_\sigma\right\}.$$

First we have

$$\|G_{\sigma,n}^{-1/2}P_\sigma G_{\sigma,n}^{-1/2}\|_{op} \leqslant \|G_{\sigma,n}^{-1}\|_{op}\|P_\sigma\|_{op} \simeq \lambda K^{2q-2}h^{-2} \simeq \lambda h^{-2q}, \text{ a.s.}$$

Then by a proof similar to that of Lemma A.1 in Xiao (2019b), we derive that $\boldsymbol{\gamma}_\sigma' P_\sigma\boldsymbol{\gamma}_\sigma = O(\lambda)$ almost surely. It follows that

$$\boldsymbol{\gamma}_\sigma' P_\sigma H_{\sigma,n}^{-1}G_\sigma H_{\sigma,n}^{-1}P_\sigma\boldsymbol{\gamma}_\sigma = O\left(\lambda\wedge\lambda^2 h^{-2q}\right) = O(\lambda^2 h_e^{-2q}), \text{ a.s.} \tag{S.5}$$

Combining equations (S.2) - (S.5),

$$\iint\{\mathbb{E}\widehat{\sigma}(s,t)-\sigma(s,t)\}^2 d_s d_t R(s,t) = O(h^{2r}) + o(h^{2p}) + O(\lambda^2 h_e^{-2q}), \text{ a.s.} \tag{S.6}$$

Now we consider the variance of $\widehat{\sigma}(s,t)$ and derive that

$$\mathrm{Var}\left\{\widehat{\sigma}(s,t)\right\} = B^{\mathsf{T}}(s,t)H_{\sigma,n}^{-1}A^{\mathsf{T}}V\mathbb{E}\left(\widehat{\boldsymbol{\sigma}}\widehat{\boldsymbol{\sigma}}^{\mathsf{T}}\right)VAH_{\sigma,n}^{-1}B(s,t).$$

Let

$$\widetilde{\Pi} = (\widetilde{\pi}_{k_1\ell_1k_2\ell_2}) = A^{\mathsf{T}}V\mathbb{E}\left(\widehat{\boldsymbol{\sigma}}\widehat{\boldsymbol{\sigma}}^{\mathsf{T}}\right)VA = \sum_{i=1}^{n} A_i^{\mathsf{T}}V_i\mathbb{E}\left(\widehat{\boldsymbol{\sigma}}_i\widehat{\boldsymbol{\sigma}}_i^{\mathsf{T}}\right)V_iA_i.$$

Then, $\mathrm{Var}\left\{\widehat{\sigma}(s,t)\right\} = B^{\mathsf{T}}(s,t)H_{\sigma,n}^{-1}\widetilde{\Pi}H_{\sigma,n}^{-1}B(s,t)$ and

$$\iint \mathrm{Var}\left\{\widehat{\sigma}(s,t)\right\}d_sd_tR(s,t) = \mathrm{tr}\left(H_{\sigma,n}^{-1}\widetilde{\Pi}H_{\sigma,n}^{-1}G_\sigma\right). \tag{S.7}$$

Since $G_\sigma \simeq h^2I$ almost surely by Lemma 6,

$$\mathrm{tr}\left(H_{\sigma,n}^{-1}\widetilde{\Pi}H_{\sigma,n}^{-1}G_\sigma\right) \simeq h^2\mathrm{tr}\left(H_{\sigma,n}^{-2}\widetilde{\Pi}\right), \text{ a.s.} \tag{S.8}$$

By Lemma 8,

$$H_{\sigma,n}^{-2} \preceq h^{-2}H^{-1}\otimes H^{-1}, \text{ a.s.}$$

where H is defined in Lemma 7. As a result,

$$\mathrm{tr}\left(H_{\sigma,n}^{-2}\widetilde{\Pi}\right) = O\left[h^{-2}\mathrm{tr}\left\{\left(H^{-1}\otimes H^{-1}\right)\widetilde{\Pi}\right\}\right], \text{ a.s.} \tag{S.9}$$

Let $\Pi = (\pi_{k_1\ell_1k_2\ell_2})$ with

$$\pi_{k_1\ell_1k_2\ell_2} = \sum_{i=1}^{n} v_i^2 \sum_{j_1\neq j_2, j_3\neq j_4} B_{k_1}(T_{ij_1})B_{\ell_1}(T_{ij_2})B_{k_2}(T_{ij_3})B_{\ell_2}(T_{ij_4}). \tag{S.10}$$

Then, by the proof between equation (S.14) and (S.18) in the supplement of Xiao (2020), we get

$$\mathrm{tr}\left\{\left(H^{-1}\otimes H^{-1}\right)\widetilde{\Pi}\right\} = O\left[\mathrm{tr}\left\{\left(H^{-1}\otimes H^{-1}\right)_+\Pi\right\}\right], \tag{S.11}$$

where for a matrix $A = (a_{ij})$, $A_+ = (|a_{ij}|)$. Note that

$$\mathrm{tr}\left\{\left(H^{-1}\otimes H^{-1}\right)_+\Pi\right\} = O_{\mathbb{P}}\left[\mathrm{tr}\left\{\left(H^{-1}\otimes H^{-1}\right)_+\mathbb{E}(\Pi)\right\}\right],$$

and combining equations (S.7) - (S.11), we have

$$\iint \mathrm{Var}\left\{\widehat{\sigma}(s,t)\right\}d_sd_tR(s,t) = O_{\mathbb{P}}\left[\mathrm{tr}\left\{\left(H^{-1}\otimes H^{-1}\right)_+\mathbb{E}(\Pi)\right\}\right]. \tag{S.12}$$

By the definition of Π in (S.10), we have the matrix format

$$\Pi = \sum_{i=1}^{n} v_i^2 \sum_{j_1 \neq j_2, j_3 \neq j_4} \{B(T_{ij_2}) \otimes B(T_{ij_1})\}\{B(T_{ij_4}) \otimes B(T_{ij_3})\}^{\mathsf{T}}$$
$$= \sum_{i=1}^{n} v_i^2 \sum_{j_1 \neq j_2, j_3 \neq j_4} \{B(T_{ij_2})B^{\mathsf{T}}(T_{ij_4})\} \otimes \{B(T_{ij_1})B^{\mathsf{T}}(T_{ij_3})\}.$$

To simplify notation, let $F_i(s) = \Lambda_0(s \wedge C_i)/\Lambda_0(C_i)$. It follows that

$$\mathbb{E}(\Pi) = \Pi_{11} + \Pi_{12} + \Pi_{21} + \Pi_{22} + \Pi_{23} + \Pi_{24} + \Pi_{31},$$

where

$$\Pi_{11} = \sum_{i=1}^{n} I(m_i \geqslant 2) v_i^2 m_i(m_i - 1) \iint \{B(t)B^{\mathsf{T}}(t)\} \otimes \{B(s)B^{\mathsf{T}}(s)\} d_s F_i(s) d_t F_i(t),$$
$$\Pi_{12} = \sum_{i=1}^{n} \frac{v_i^2 I(m_i \geqslant 2)}{m_i(m_i - 1)} \iint \{B(t)B^{\mathsf{T}}(s)\} \otimes \{B(s)B^{\mathsf{T}}(t)\} d_s F_i(s) d_t F_i(t),$$
$$\Pi_{21} = \sum_{i=1}^{n} I(m_i \geqslant 3) v_i^2 m_i(m_i - 1)(m_i - 2)$$
$$\times \iiint \{B(t_1)B^{\mathsf{T}}(t_2)\} \otimes \{B(s)B^{\mathsf{T}}(s)\} d_s F_i(s) d_{t_1} F_i(t_1) d_{t_2} F_i(t_2),$$
$$\Pi_{22} = \sum_{i=1}^{n} I(m_i \geqslant 3) v_i^2 m_i(m_i - 1)(m_i - 2)$$
$$\times \iiint \{B(t_1)B^{\mathsf{T}}(s)\} \otimes \{B(s)B^{\mathsf{T}}(t_2)\} d_s F_i(s) d_{t_1} F_i(t_1) d_{t_2} F_i(t_2),$$
$$\Pi_{23} = \sum_{i=1}^{n} I(m_i \geqslant 3) v_i^2 m_i(m_i - 1)(m_i - 2)$$
$$\times \iiint \{B(s)B^{\mathsf{T}}(s)\} \otimes \{B(t_1)B^{\mathsf{T}}(t_2)\} d_s F_i(s) d_{t_1} F_i(t_1) d_{t_2} F_i(t_2),$$
$$\Pi_{24} = \sum_{i=1}^{n} I(m_i \geqslant 3) v_i^2 m_i(m_i - 1)(m_i - 2)$$
$$\times \iiint \{B(s)B^{\mathsf{T}}(t_1)\} \otimes \{B(t_2)B^{\mathsf{T}}(s)\} d_s F_i(s) d_{t_1} F_i(t_1) d_{t_2} F_i(t_2),$$
$$\Pi_{31} = \sum_{i=1}^{n} I(m_i \geqslant 4) v_i^2 m_i(m_i - 1)(m_i - 2)(m_i - 3)$$
$$\times \iiiint \{B(s_1)B^{\mathsf{T}}(t_1)\} \otimes \{B(s_2)B^{\mathsf{T}}(t_2)\} d_{s_1} F_i(s_1) d_{s_2} F_i(s_2) d_{t_1} F_i(t_1) d_{t_2} F_i(t_2).$$

As $v_i = \{nm_i(m_i-1)\}^{-1}$, it is easy to verify the following:

$$I(m_i \geqslant 2)v_i^2 m_i(m_i-1) \leqslant n^{-2},$$

$$I(m_i \geqslant 3)v_i^2 m_i(m_i-1)(m_i-2) \leqslant n^{-2},$$

$$I(m_i \geqslant 4)v_i^2 m_i(m_i-1)(m_i-2)(m_i-3) \leqslant n^{-2}.$$

Thus, we have $\Pi_{ij} \leqslant \widetilde{\Gamma}_{ij}$, where

$$\widetilde{\Gamma}_{11} = n^{-1}\iint \{B(t)B^{\mathsf{T}}(t)\} \otimes \{B(s)B^{\mathsf{T}}(s)\} d_s d_t \left\{ n^{-1}\sum_{i=1}^{n} F_i(s)F_i(t) \right\},$$

$$\widetilde{\Gamma}_{12} = n^{-1}\iint \{B(t)B^{\mathsf{T}}(s)\} \otimes \{B(s)B^{\mathsf{T}}(t)\} d_s d_t \left\{ n^{-1}\sum_{i=1}^{n} F_i(s)F_i(t) \right\},$$

$$\Gamma_{21} = n^{-1}\iiint \{B(t_1)B^{\mathsf{T}}(t_2)\} \otimes \{B(s)B^{\mathsf{T}}(s)\} d_s d_{t_1} d_{t_2} \left\{ n^{-1}\sum_{i=1}^{n} F_i(s)F_i(t_1)F_i(t_2) \right\},$$

$$\Gamma_{22} = n^{-1}\iiint \{B(t_1)B^{\mathsf{T}}(s)\} \otimes \{B(s)B^{\mathsf{T}}(t_2)\} d_s d_{t_1} d_{t_2} \left\{ n^{-1}\sum_{i=1}^{n} F_i(s)F_i(t_1)F_i(t_2) \right\},$$

$$\widetilde{\Gamma}_{23} = n^{-1}\iiint \{B(s)B^{\mathsf{T}}(s)\} \otimes \{B(t_1)B^{\mathsf{T}}(t_2)\} d_s d_{t_1} d_{t_2} \left\{ n^{-1}\sum_{i=1}^{n} F_i(s)F_i(t_1)F_i(t_2) \right\},$$

$$\widetilde{\Gamma}_{24} = n^{-1}\iiint \{B(s)B^{\mathsf{T}}(t_1)\} \otimes \{B(t_2)B^{\mathsf{T}}(s)\} d_s d_{t_1} d_{t_2} \left\{ n^{-1}\sum_{i=1}^{n} F_i(s)F_i(t_1)F_i(t_2) \right\},$$

$$\widetilde{\Gamma}_{31} = n^{-1}\iiiint \{B(s_1)B^{\mathsf{T}}(t_1)\} \otimes \{B(s_2)B^{\mathsf{T}}(t_2)\} d_{s_1} d_{s_2} d_{t_1} d_{t_2} \times \left\{ n^{-1}\sum_{i=1}^{n} F_i(s_1)F_i(s_2)F_i(t_1)F_i(t_2) \right\}.$$

Let

$$\widetilde{\Gamma} = \widetilde{\Gamma}_{11} + \widetilde{\Gamma}_{12} + \widetilde{\Gamma}_{21} + \widetilde{\Gamma}_{22} + \widetilde{\Gamma}_{23} + \widetilde{\Gamma}_{24} + \widetilde{\Gamma}_{31},$$

It follows by equation (S.12) that

$$\iint \mathrm{Var}\{\widehat{\sigma}(s,t)\}\, d_s d_t R(s,t) = O_{\mathbb{P}}\left[\mathrm{tr}\left\{\left(H^{-1} \otimes H^{-1}\right)_+ \widetilde{\Gamma}\right\}\right]. \tag{S.13}$$

Now we can define Γ_{ij} where the empirical CDFs in defining $\widetilde{\Gamma}_{ij}$ are replaced by their expectation with the censoring times. For example, $n^{-1}\sum_{i=1}^{n} F_i(s)F_i(t)$ in $\widetilde{\Gamma}_{11}$ is replaced

by $\mathbb{E}\{F_1(s)F_1(t)\}$. We also define Γ, similar to $\widetilde{\Gamma}$. Then, we derive from (S.13) that

$$\iint \mathrm{Var}\left\{\widehat{\sigma}(s,t)\right\} d_s d_t R(s,t) = O_{\mathbb{P}}\left[\mathrm{tr}\left\{\left(H^{-1}\otimes H^{-1}\right)_+ \Gamma\right\}\right]. \tag{S.14}$$

By Assumption 8, those CDFs in defining Γ_{ij}s have partial derivatives that are bounded away from 0 and infinity. Then, (S.14) still holds when we replace the CDFs in defining Γ_{ij}s by the proper CDFs corresponding to uniform distribution in the unit cube. Then the rest of proof can be carried out similar to the that for equation (S.28) in the supplement of Xiao (2020) and hence is omitted.

Finally, by combining (S.6) and (S.14), we derive that

$$\iint \mathbb{E}\left\{\widehat{\sigma}(s,t)-\sigma(s,t)\right\}^2 d_s d_t R(s,t) = O(h^{2r}) + o(h^{2p}) + O(\lambda^2 h_e^{-2q}) + O_{\mathbb{P}}(nh_e^{-2}).$$

By Assumption 4, $\partial^2 R(s,t)/\partial s \partial t$ is bounded away from 0 and infinity, hence

$$\iint \mathbb{E}\left\{\widehat{\sigma}(s,t)-\sigma(s,t)\right\}^2 \mathrm{d}s\,\mathrm{d}t \simeq \iint \mathbb{E}\left\{\widehat{\sigma}(s,t)-\sigma(s,t)\right\}^2 d_s d_t R(s,t),$$

and the proof is complete.

In Lemmas 5-8, we assume Assumptions 1-8 always hold.

LEMMA 5: *Let* $\widetilde{\tau} = \sum_{i=1}^n m_i(m_i-1)v_i$ *with* $v_i = \{nm_i(m_i-1)\}^{-1}$ *and*

$$\widetilde{R}_n(s,t) = \widetilde{\tau}^{-1}\sum_{i=1}^n v_i \sum_{j_1\neq j_2} I(T_{ij_1}\leqslant s)I(T_{ij_2}\leqslant t)$$

be an empirical cumulative distribution function. Let $\widetilde{Z}(s,t) = \widetilde{R}_n(s,t) - R(s,t)$, *where* $R(s,t)$ *is defined in Lemma 1. Then,* $\|\widetilde{Z}\| = o(h^2)$ *almost surely.*

LEMMA 6: *Let* $G_\sigma = \iint\{B(s)B^{\mathsf{T}}(s)\}\otimes\{B(t)B^{\mathsf{T}}(t)\}d_s d_t R(s,t)$, *where* $R(s,t)$ *is defined in Lemma 1. Then,*

$$G_{\sigma,n} \simeq G_\sigma \simeq h^2 I, \text{ a.s.}$$

Proof of 6. It can be shown that

$$G_{\sigma,n} = \iint\{B(s)B^{\mathsf{T}}(s)\}\otimes\{B(t)B^{\mathsf{T}}(t)\}d_s d_t \widetilde{R}_n(s,t),$$

where $\widetilde{R}_n(s,t)$ is defined in Lemma 5.

LEMMA 7: *Let* $G = \int B(s)B^{\mathsf{T}}(s)d_sQ(s)$, *where* $Q(s)$ *is defined in Lemma 1. Also let* $H = G + \lambda P$. *Then,* $G \simeq hI$ *and* $\|H\|_\infty = h_e^{-1}$.

Proof of 7. The lemma is just parts of Lemmas A.3 and A.6 in Xiao (2020).

LEMMA 8: *Let* H *be as in Lemma 7. Then,*

$$H_{\sigma,n}^{-2} \preceq h^{-2}H^{-1} \otimes H^{-1}, \text{ a.s.}$$

Proof of 8. We derive that

$$H_{\sigma,n} = G_{\sigma,n} + P_\sigma \simeq h^2 I + \lambda h(I \otimes P + P \otimes I).$$

Thus,

$$\begin{aligned} H_{\sigma,n}^2 &\simeq \left\{h^2 I + \lambda h(I \otimes P + P \otimes I)\right\}\left\{h^2 I + \lambda h(I \otimes P + P \otimes I)\right\} \\ &\geqslant h^2(G + \lambda P) \otimes (G + \lambda P). \end{aligned}$$

It follows that

$$H_{\sigma,n}^{-2} \preceq h^{-2}H^{-1} \otimes H^{-1},$$

which proves the lemma.

Web Appendix C. Proofs of Assumptions 4 and 8 for Random Censoring

We shall generate the censoring times C_i randomly according to the following cumulative distribution function

$$F_C(t) = \delta_1 I(t = 1) + \frac{1-\delta_1}{1-\delta_2}(t - \delta_2)I(\delta_2 \leqslant t \leqslant 1),$$

where δ_1 and δ_2 are two fixed constants in $(0, 1)$. The mixed censoring simulation setting in Section 4 is a special case in which $\delta_1 = \delta_2 = 0.2$. We derive that

$$\rho_1(t) = \delta_1 \Lambda_0^{(1)}(t) + \frac{1-\delta_1}{1-\delta_2}\int_{\delta_2}^{1} \frac{\Lambda_0^{(1)}(t \wedge c)}{\Lambda_0(c)}\,\mathrm{d}c,$$

which is continuous. Then we have $\rho_1(t) \geqslant \delta_1 \Lambda_0^{(1)}(t)$, which is bounded away from 0 because by Assumption 3, $\Lambda_0^{(1)}(t)$ is bounded away from 0. We also have

$$\begin{aligned}
\rho_1(t) &\leqslant \delta_1 \Lambda_0^{(1)}(t) + \frac{1-\delta_1}{1-\delta_2} \int_{\delta_2}^{1} \frac{\Lambda_0^{(1)}(t \wedge c)}{\Lambda_0(c)} \,\mathrm{d}c \\
&\leqslant \delta_1 \Lambda_0^{(1)}(t) + \frac{1-\delta_1}{1-\delta_2} \int_{\delta_2}^{1} \frac{\|\Lambda_0^{(1)}\|}{\Lambda_0(\delta_2)} \,\mathrm{d}c \\
&\leqslant \delta_1 \|\Lambda_0^{(1)}\| + (1-\delta_1) \frac{\|\Lambda_0^{(1)}\|}{\Lambda_0(\delta_2)}.
\end{aligned}$$

Hence, ρ_1 is also bounded away from infinity.

Next we consider ρ_2. We have

$$\rho_2(s,t) = \delta_1 \Lambda_0^{(1)}(s) \Lambda_0^{(1)}(t) + \frac{1-\delta_1}{1-\delta_2} \int_{\delta_2}^{1} \frac{\Lambda_0^{(1)}(s \wedge x) \Lambda_0^{(1)}(t \wedge x)}{\Lambda_0^2(x)} \,\mathrm{d}x$$

and the rest of the derivations are similar to the above.

Similarly we could show that ρ_3 and ρ_4 are also continuous and bounded away from 0 and infinity.

Table S.1
Median (interquartile range) of integrated squared error for estimating the mean function using penalized splines with both OBS and SUBJ weights.

			Gamma					
			No censoring		Mixed censoring		Uniform censoring	
ρ	n	$\sigma^2_{Z^*}$	OBS	SUBJ	OBS	SUBJ	OBS	SUBJ
0.00	150	0.5	0.000 (0.000)	0.000 (0.000)	0.000 (0.000)	0.000 (0.000)	0.001 (0.001)	0.001 (0.001)
		1.0	0.000 (0.000)	0.000 (0.000)	0.000 (0.000)	0.000 (0.000)	0.000 (0.001)	0.000 (0.001)
	300	0.5	0.000 (0.000)	0.000 (0.000)	0.000 (0.000)	0.000 (0.000)	0.000 (0.000)	0.000 (0.000)
		1.0	0.000 (0.000)	0.000 (0.000)	0.000 (0.000)	0.000 (0.000)	0.000 (0.000)	0.000 (0.000)
0.50	150	0.5	0.011 (0.017)	0.001 (0.002)	0.022 (0.021)	0.002 (0.002)	0.013 (0.067)	0.007 (0.016)
		1.0	0.059 (0.060)	0.002 (0.002)	0.068 (0.039)	0.004 (0.004)	0.082 (0.077)	0.006 (0.013)
	300	0.5	0.016 (0.008)	0.000 (0.000)	0.011 (0.007)	0.001 (0.002)	0.020 (0.019)	0.003 (0.009)
		1.0	0.069 (0.037)	0.001 (0.001)	0.073 (0.042)	0.003 (0.003)	0.059 (0.101)	0.006 (0.010)
0.85	150	0.5	0.171 (0.109)	0.003 (0.003)	0.102 (0.116)	0.007 (0.010)	0.123 (0.102)	0.021 (0.029)
		1.0	0.557 (0.467)	0.007 (0.009)	0.409 (0.343)	0.016 (0.038)	0.322 (0.706)	0.046 (0.081)
	300	0.5	0.141 (0.056)	0.001 (0.002)	0.102 (0.057)	0.005 (0.008)	0.120 (0.096)	0.014 (0.011)
		1.0	0.596 (0.184)	0.003 (0.003)	0.593 (0.297)	0.011 (0.014)	0.448 (0.528)	0.019 (0.025)
			Lognormal					
			No censoring		Mixed censoring		Uniform censoring	
ρ	n	$\sigma^2_{Z^*}$	OBS	SUBJ	OBS	SUBJ	OBS	SUBJ
0.00	150	0.5	0.000 (0.000)	0.000 (0.000)	0.000 (0.000)	0.000 (0.000)	0.000 (0.000)	0.000 (0.000)
		1.0	0.000 (0.000)	0.000 (0.000)	0.000 (0.000)	0.000 (0.000)	0.000 (0.001)	0.000 (0.001)
	300	0.5	0.000 (0.000)	0.000 (0.000)	0.000 (0.000)	0.000 (0.000)	0.000 (0.000)	0.000 (0.001)
		1.0	0.000 (0.000)	0.000 (0.000)	0.000 (0.000)	0.000 (0.000)	0.000 (0.000)	0.000 (0.000)
0.50	150	0.5	0.016 (0.024)	0.001 (0.002)	0.012 (0.027)	0.002 (0.003)	0.023 (0.030)	0.008 (0.012)
		1.0	0.073 (0.120)	0.001 (0.002)	0.075 (0.081)	0.005 (0.005)	0.065 (0.192)	0.013 (0.019)
	300	0.5	0.017 (0.014)	0.000 (0.000)	0.017 (0.017)	0.001 (0.001)	0.019 (0.016)	0.002 (0.005)
		1.0	0.048 (0.061)	0.001 (0.001)	0.054 (0.078)	0.002 (0.003)	0.064 (0.081)	0.003 (0.009)
0.85	150	0.5	0.141 (0.048)	0.003 (0.004)	0.098 (0.137)	0.009 (0.008)	0.079 (0.164)	0.027 (0.023)
		1.0	0.477 (0.344)	0.006 (0.009)	0.297 (0.684)	0.014 (0.012)	0.422 (0.450)	0.041 (0.046)
	300	0.5	0.102 (0.052)	0.001 (0.002)	0.107 (0.091)	0.005 (0.005)	0.143 (0.150)	0.013 (0.014)
		1.0	0.579 (0.348)	0.003 (0.003)	0.375 (0.283)	0.005 (0.009)	0.420 (0.611)	0.010 (0.015)

Web Appendix D. Additional Simulation Results

The mean function simulation results for all simulation settings are summarized in in Table S.1, and the covariance function simulation results are summarized in Table S.2. Figure S.1 and Figure S.2 plot the averages of the estimated mean functions from penalized splines with either OBS or SUBJ weights for all simulation settings with informative observations.

Web Appendix E. Additional Data Analysis Results

The estimated correlation and variance functions, using both the SUBJ and OBS weights are displayed in Figure S.3.

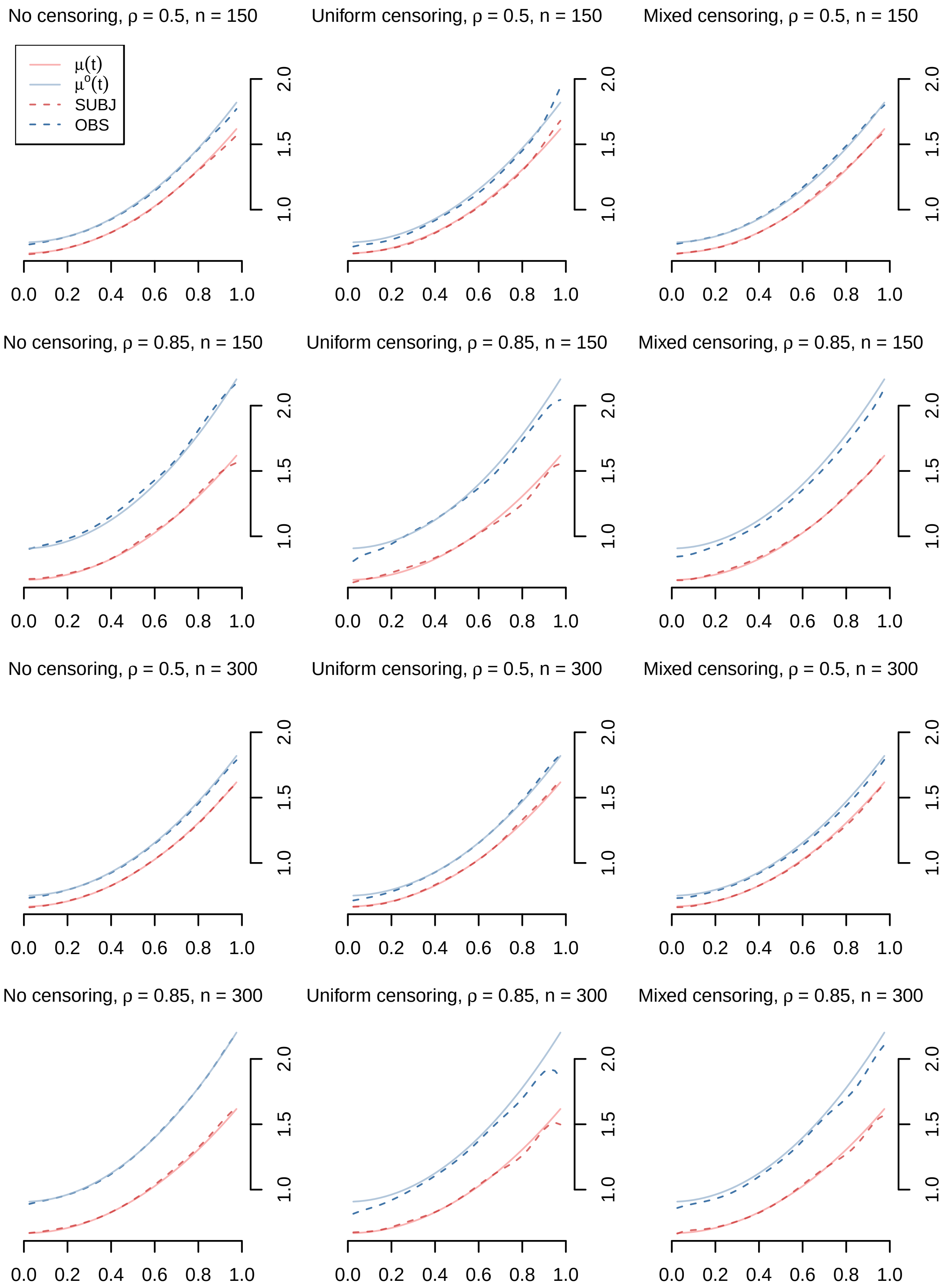

Figure S.1: Average of 200 estimates of the mean function using penalized splines with both OBS and SUBJ weights for simulation settings in which the frailty variable Z_i^* follows a gamma distribution. The true mean function is $\mu(t) = t^2 + 2/3$ and $\mu^o(t)$ is the formula in (4) associated with equal weights, specifically $\mu^o(t) = (t^2 + 2/3)(1 + \rho^2\sigma^2_{Z^*})$.

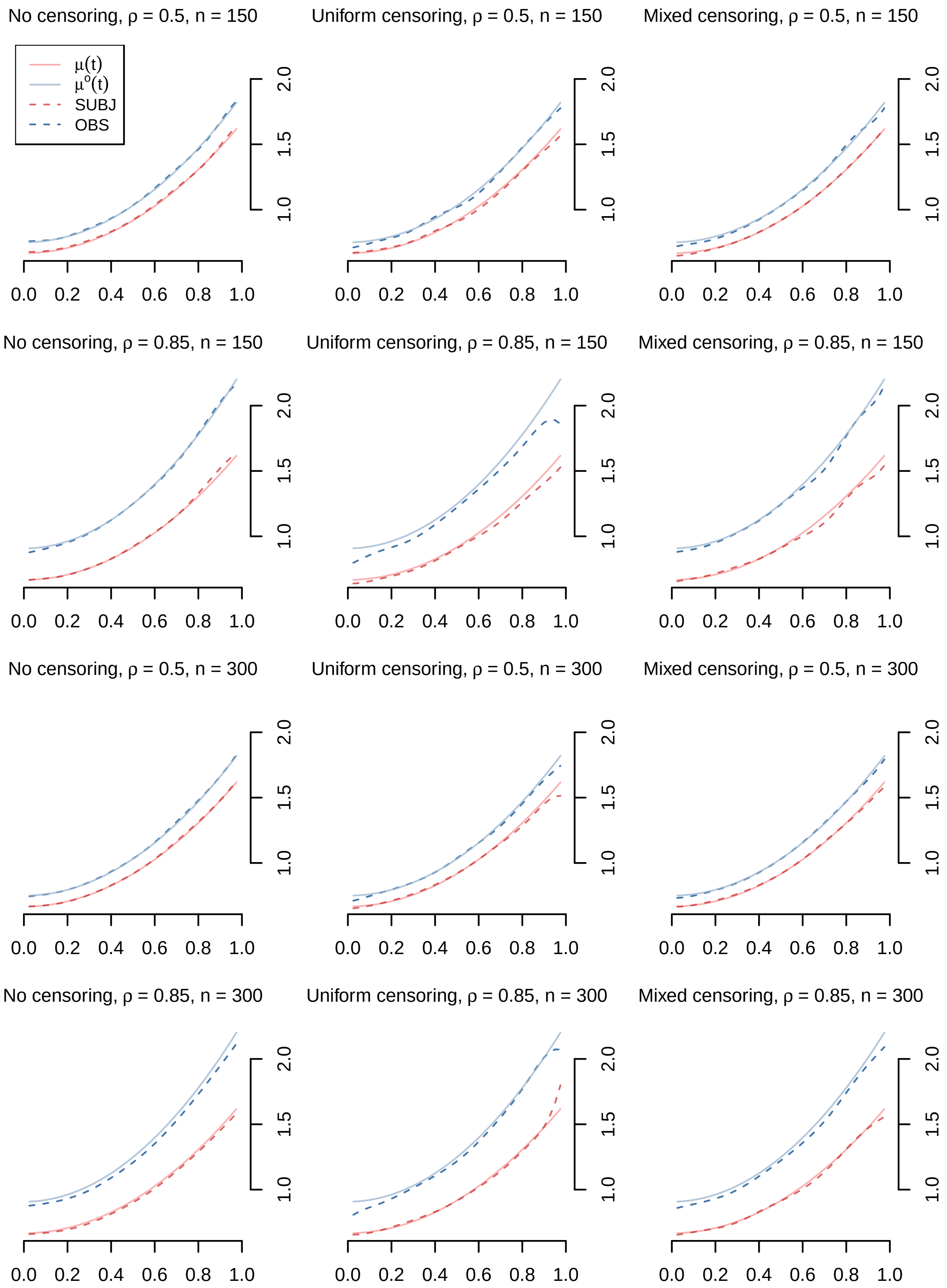

Figure S.2: Average of 200 estimates of the mean function using penalized splines with both OBS and SUBJ weights for simulation settings in which the frailty variable Z_i^* follows a lognormal distribution. The true mean function is $\mu(t) = t^2 + 2/3$ and $\mu^o(t)$ is the formula in (4) associated with equal weights, specifically $\mu^o(t) = (t^2 + 2/3)(1 + \rho^2\sigma^2_{Z^*})$.

Table S.2

Median (interquartile range) of integrated squared error for estimating the covariance function using penalized splines with both OBS and SUBJ weights.

			Gamma					
			No censoring		Mixed censoring		Uniform censoring	
ρ	n	$\sigma^2_{Z^*}$	OBS	SUBJ	OBS	SUBJ	OBS	SUBJ
0.00	150	0.5	0.000 (0.000)	0.000 (0.000)	0.000 (0.000)	0.000 (0.000)	0.000 (0.000)	0.000 (0.000)
		1.0	0.000 (0.000)	0.000 (0.000)	0.000 (0.000)	0.000 (0.000)	0.000 (0.000)	0.000 (0.000)
	300	0.5	0.000 (0.000)	0.000 (0.000)	0.000 (0.000)	0.000 (0.000)	0.000 (0.000)	0.000 (0.000)
		1.0	0.000 (0.000)	0.000 (0.000)	0.000 (0.000)	0.000 (0.000)	0.000 (0.000)	0.000 (0.000)
0.50	150	0.5	0.018 (0.015)	0.000 (0.001)	0.011 (0.066)	0.001 (0.002)	0.005 (0.013)	0.003 (0.006)
		1.0	0.224 (0.653)	0.004 (0.009)	0.242 (1.141)	0.008 (0.014)	0.161 (0.451)	0.008 (0.020)
	300	0.5	0.009 (0.010)	0.000 (0.000)	0.006 (0.041)	0.001 (0.001)	0.011 (0.033)	0.001 (0.002)
		1.0	0.220 (0.472)	0.003 (0.004)	0.211 (0.789)	0.005 (0.005)	0.148 (0.567)	0.011 (0.017)
0.85	150	0.5	0.324 (0.581)	0.008 (0.014)	0.033 (0.226)	0.011 (0.018)	0.044 (0.400)	0.017 (0.014)
		1.0	5.359 (21.07)	0.043 (0.157)	5.568 (22.08)	0.077 (0.049)	0.407 (4.671)	0.129 (0.092)
	300	0.5	0.235 (0.399)	0.003 (0.003)	0.068 (0.577)	0.006 (0.013)	0.032 (0.129)	0.007 (0.019)
		1.0	3.022 (15.30)	0.045 (0.060)	2.556 (5.849)	0.054 (0.059)	2.950 (10.06)	0.070 (0.076)

			Lognormal					
			No censoring		Mixed censoring		Uniform censoring	
ρ	n	$\sigma^2_{Z^*}$	OBS	SUBJ	OBS	SUBJ	OBS	SUBJ
0.00	150	0.5	0.000 (0.000)	0.000 (0.000)	0.000 (0.000)	0.000 (0.000)	0.000 (0.000)	0.000 (0.000)
		1.0	0.000 (0.000)	0.000 (0.000)	0.000 (0.000)	0.000 (0.000)	0.000 (0.000)	0.000 (0.000)
	300	0.5	0.000 (0.000)	0.000 (0.000)	0.000 (0.000)	0.000 (0.000)	0.000 (0.000)	0.000 (0.000)
		1.0	0.000 (0.000)	0.000 (0.000)	0.000 (0.000)	0.000 (0.000)	0.000 (0.000)	0.000 (0.000)
0.50	150	0.5	0.032 (0.109)	0.001 (0.002)	0.011 (0.160)	0.002 (0.004)	0.022 (0.251)	0.004 (0.006)
		1.0	2.885 (7.116)	0.011 (0.043)	0.341 (1.786)	0.012 (0.028)	0.801 (3.151)	0.027 (0.071)
	300	0.5	0.093 (0.113)	0.001 (0.002)	0.043 (0.088)	0.001 (0.002)	0.025 (0.124)	0.003 (0.005)
		1.0	0.614 (2.761)	0.005 (0.006)	0.747 (12.21)	0.010 (0.021)	0.778 (9.974)	0.013 (0.019)
0.85	150	0.5	0.691 (1.745)	0.007 (0.011)	0.652 (5.084)	0.031 (0.042)	0.156 (0.257)	0.032 (0.026)
		1.0	12.37 (74.19)	0.034 (0.106)	9.179 (96.96)	0.176 (0.238)	9.697 (29.30)	0.137 (0.254)
	300	0.5	0.550 (1.728)	0.004 (0.005)	0.562 (2.247)	0.009 (0.021)	0.941 (3.214)	0.024 (0.017)
		1.0	32.76 (93.44)	0.076 (0.133)	10.48 (129.7)	0.060 (0.078)	15.32 (71.81)	0.124 (0.244)

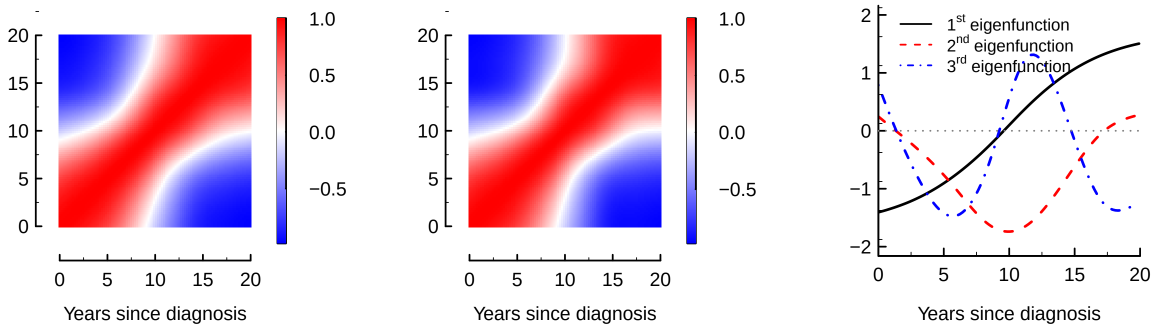

Figure S.3: (Left) Estimated correlation function using both the SUBJ weights. (Center) Estimated correlation function using both the OBS weights. (Right) First three estimated eigenfunctions using the OBS weights.

.

References

Xiao, L. (2019a). Asymptotic theory of penalized splines. *Electronic Journal of Statistics* **13,** 747–794.

Xiao, L. (2019b). Asymptotics of bivariate penalised splines. *Journal of Nonparametric Statistics* **31,** 289–314.

Xiao, L. (2020). Asymptotic properties of penalized splines for functional data. *Bernoulli* **26,** 2847–2875.
